# Exploring Body Image Awareness With a Large Language Model–Based Conversational Agent: Qualitative Study With Young Adults

**DOI:** 10.2196/78829

**Published:** 2025-11-17

**Authors:** Xuan Zhang, Ahmed Zayed, Josefin Rehn Hamrin, Arzu Güneysu, Sanna Kuoppamäki

**Affiliations:** 1 Department of Biomedical Engineering and Health Systems KTH Royal Institute of Technology Huddinge Sweden; 2 Department of Public Health and Primary Care KU Leuven Leuven Belgium; 3 Department of Computing Science Umeå University Umeå Sweden

**Keywords:** AI agents, body image, conversational agents, qualitative study, young adults

## Abstract

**Background:**

Body image plays a crucial role in both physical and mental health, influencing self-esteem, eating behaviors, and psychological well-being. Young adults are particularly vulnerable to body dissatisfaction, defined as negative thoughts or feelings about one’s appearance. The benefits of positive body image, characterized by body appreciation and acceptance, are widely recognized, but few digital interventions are designed to support it for young adults.

**Objective:**

We designed a conversational artificial intelligence (AI) agent integrating biomedical information on eating disorders and the principles of cognitive behavioral therapy to enable open-domain conversations on body image. The study explores young adults’ strategies to maintain a positive body image without the agent, the characteristics of conversations with the agent, and the advantages and drawbacks of having a conversation for body image concerns.

**Methods:**

A qualitative study consisting of in-depth interviews with young adults was conducted among 15 young adults (aged 20-30 years) who used the AI agent in their homes for a 1-week period. Data comprise preinterviews exploring young adults’ maintenance of body image without the AI agent, text-based conversations with an AI agent (n=933 messages), and postinterviews on the perceived impact of conversations on body image awareness. Interview transcripts were analyzed through thematic analysis. Content analysis was applied to analyze the conversations with the AI agent.

**Results:**

Young adults’ body image awareness was connected to self-acceptance, confidence, and valuing body functionality. Participants used several strategies to maintain body image without the AI agent, ranging from social support networks to exercise and positive self-talk. The conversations with the AI agent were categorized into (1) body image awareness, (2) body image–related eating and behavioral regulation, (3) body-focused mindfulness, and (4) social conversation with the agent. Three themes of perceived advantages and drawbacks regarding the conversations with the agent were identified as (1) facilitating body image awareness and self-reflection, (2) availability of conversational support, and (3) discontinuities in user engagement.

**Conclusions:**

Young adults’ body image awareness is closely linked to self-acceptance and self-appreciation. In this context, the AI agent was perceived as an available, accessible, and nonjudgmental conversational support in raising body image awareness through self-reflection and self-compassion. Challenges remain in sustaining long-term user engagement, which address the need for multidimensional personalization of the agent.

## Introduction

### Overview

Body image, defined as a person’s perceptions, thoughts, and feelings about their body and physical appearance, is associated with physical and mental health [1]. Young adults with positive body image report higher levels of self-esteem, better coping strategies, and healthier eating behaviors [2]. Conversely, body dissatisfaction, defined as a negative attitude toward one’s physical appearance, is strongly associated with disordered eating patterns, depression, anxiety, and reduced physical activity [3].

Conversational agents driven by natural language processing and large language models (LLMs) provide new possibilities to support body image through simulating human-like conversations [4]. These technologies are used in preventative health to provide health information and guidance, aid symptom assessment, enhance patient education, and support disease management [5]. The central part is their ability to comprehend, analyze, and reply to user questions with information in a conversational format that mimics human dialogue. Conversational artificial intelligence (AI) technologies can facilitate connectivity between health care providers and patients [6], which is expected to alleviate strain on health care systems by automating routine tasks [7]. These technologies are increasingly used as a part of preventative mental health, instead of as a medical intervention [8,9].

In this study, we designed and evaluated an LLM-based conversational AI agent to be used in open-domain conversations for body image. The study explored young adults’ strategies to maintain a positive body image without the agent, the characteristics of conversations with the agent, and the advantages and drawbacks of having a conversation for body image concerns. We designed a text-based conversational AI agent integrating biomedical information on eating disorders and the principles of cognitive behavioral therapy (CBT) to enable anonymous conversations on body image. The agent enabled the user to ask open-domain questions on body image and eating behaviors, and it provided answers on information about eating disorders and suggestions for identification and reframing of negative thoughts. Through an iterative prototype development, we aimed to incorporate key aspects of positive body image in agent’s responses, including body appreciation, body acceptance, inner positivity, and interpreting information in a body-protective manner [10].

Previous studies on chatbots for body image have been investigated in specific psychiatric conditions in clinical settings [11]. Our study is among the first to explore the role of an LLM-based agent in assisting young adults in self-care and open conversational setting rather than in a strictly clinical framework. Existing digital interventions for promoting positive body image are typically structured, static, and delivered in predefined modules or exercises [12,13]. In contrast, conversational AI agents offer an interactive, responsive, and user-driven format that adapts to individual needs and contexts. Such agents may support self-reflection through dialogue, allow immediate access to supportive conversations, and provide a private space for exploring sensitive body image concerns. These affordances make chat-based interventions particularly suitable for young adults, who frequently use digital communication and may prefer conversational engagement over didactic formats. Thus, this study aims to explore how a conversational agent might complement existing approaches by offering continuous and dialogic support for body image awareness.

To fulfill the research gaps above, we report findings from a qualitative interview study conducted with 15 young adults (aged 20-30 years) who used an LLM-based agent for a 1-week period in their homes. The study design did not serve any diagnostic or therapeutic purpose; instead, it aimed to explore the feasibility and acceptability of using an AI agent to support body positivity in everyday contexts. The research questions (RQs) are the following:

RQ1: In what way young adults maintain their body image without the conversation with an AI agent?

RQ2: What kind of open-domain conversations young adults have with the AI agent on body image?

RQ3: What are the advantages and drawbacks of using an AI agent for conversations aimed at enhancing body image awareness?

### Background

Body image is a multidimensional concept that includes cognitive, affective, and behavioral aspects of thoughts and feelings toward one’s body [1,14]. Body image is associated with eating behaviors and other body-related actions related to physical appearance, body acceptance, and self-care [10,15]. In this study, we use the term “body image awareness” to encompass the cognitive and affective dimensions of body image [16]. This concept is closely associated with positive body image, defined by Tylka and Wood-Barcalow [10], which consists of body appreciation, body acceptance, inner positivity, and interpreting information in a body-protective manner. Conceptually, body image awareness is connected to body image satisfaction and dissatisfaction. Body image dissatisfaction indicates a negative attitude toward one’s own physical appearance and is assumed to originate from a perceived discrepancy between the actual physical appearance (actual body image) and the desired ideal state of the body (ideal body image) [17]. Therefore, body image can be defined as a mental representation of one’s body that is influenced by the physical characteristics (eg, body size or shape), psychological characteristics (eg, perfectionism or low self-esteem), and the sociocultural context (eg, cultural ideal of beauty) [18].

Body image awareness can be enhanced by various types of technologies, ranging from wearables to self-tracking devices [16,19]. Conversational agents have been developed to facilitate positive body image or treat eating disorders. Beilharz et al [12] designed KIT, a prototype based on a conversation decision tree that offered psychoeducational information on body image and eating disorders and evidence-based coping strategies. Matheson et al [13] conducted a randomized controlled trial among adolescents to assess the use of a body image chatbot as an intervention to protect against the negative exposures associated with social media. Both studies reported positive feedback in the chatbot use among young adults and minor improvements in body image concerns after using the chatbot. However, these studies did not integrate LLMs into the development of the chatbot, and they did not consider the diversity of conversations that young adults could have with the agent.

Conversational AI agents have been used as a therapeutic self-help tool for young adults experiencing symptoms of depression. Fitzpatrick [7] examined the effectiveness of Woebot, a conversational agent designed to deliver CBT-based self-help for young adults experiencing symptoms of depression and anxiety through simulating therapeutic conversations. The therapeutic content delivered by Woebot was grounded in CBT principles, through which users were guided to identify and challenge negative thought patterns. This involved asking users to consider alternative interpretations of situations that evoked negative emotions. Through this process, users learned to question the accuracy of their negative thoughts and to replace them with balanced perspectives, a technique known as cognitive restructuring [20]. Participants who interacted with Woebot experienced notable decreases in depression and anxiety symptoms when compared to participants provided with a digital CBT e-book.

Previous studies on chatbots for body image awareness have been limited to rule-based architecture, which provides limited capabilities for personalization or continuous conversations. Additionally, most existing interventions have focused on eating disorders or specific psychiatric conditions, often serving clinical settings rather than self-care [11,13]. Our study is among the first to explore the role of an AI agent in assisting young adults in self-care and open conversational setting rather than in a strictly clinical framework. Our study focuses primarily on body image awareness, which we define as attitudinal and affective dimensions of body image, such as one’s thoughts and feelings of body satisfaction and appearance-related self-evaluation.

## Methods

### Study Design

This study used a qualitative research design to explore the perceived impact of a conversational AI agent on young adults’ body image awareness. We developed a prototype of an LLM-based conversational agent, referred to as TrueBalance, which was explored with 15 young adults aged 20-30 years through a qualitative interview study. The study was structured into the following three phases: (1) the preinteraction phase, including semistructured interviews and a background questionnaire before interaction with the agent; (2) interaction with the agent in a home environment for a 1-week period; and (3) the postinteraction phase, including semistructured interviews and a background questionnaire after the interaction with the agent.

In the preinteraction phase, we conducted semistructured interviews with participants to explore their baseline attitudes toward body image, including their personal definitions, existing coping strategies, and expectations regarding conversational technologies. The Body Image Satisfaction Questionnaire [21] was administered to supplement the interview data and provide contextual insights into participants’ self-perceptions. Before the interaction phase, participants were given verbal and written instructions for interacting with the agent. Participants were asked to use an anonymous ID before starting the conversation with the agent. They were advised to interact with the agent a minimum of 3 times during a 7-day period and recommended to use it for 15-30 minutes per session. They were told that they can stop the conversation at any time and continue it later. This explorative research design emphasized self-directed interaction to allow participants to form authentic and situated impressions of the agent.

In the postinteraction phase, participants took part in a second stage of semistructured interviews, which explored potential changes in their body image and their experiences with TrueBalance regarding emotional engagement and the relational dynamics. Postinteraction questionnaires were used to supplement and contextualize the qualitative findings.

Participants’ written interactions with the TrueBalance were collected and analyzed as part of the qualitative dataset. These chat transcripts served as a supplementary data source to contextualize participants’ reflections and to examine patterns of engagement, thematic content, and emotional tone during AI-mediated conversations. This approach enabled data triangulation and a deeper understanding of how participants interacted with the agent in relation to their body image concerns. This study is reported in accordance with the Standards for Reporting Qualitative Research guidelines [22].

### TrueBalance: Development of an LLM-Based Conversational AI Agent

#### Knowledge Base for Evidence-Based Guidance

The first phase of prototype development involved a comprehensive literature review to establish the agent’s knowledge base (Multimedia Appendix 1). This review integrated evidence on the biomedical and psychological determinants of eating disorders, including how stress-related hormonal fluctuations can influence emotional eating. For instance, the agent could explain that heightened stress may lead to increased body dissatisfaction, helping users understand these experiences as complex biopsychosocial processes rather than personal shortcomings. Additionally, core CBT principles were integrated as a conversational strategy, ensuring that interactions were structured to support users in self-reflection and cognitive restructuring.

An initial prototype of TrueBalance was developed using OpenAI’s GPT-3.5 model via the custom GPT Builder. Prompt engineering was applied to craft the instructions, ensuring that responses adhered to CBT principles (Multimedia Appendix 2). The initial prototype was tested through a pilot study with 3 participants, which revealed developmental areas for personalization, processing of contextual information, and maintaining anonymity in conversation logs.

#### Refinement and Automation Architecture

**Figure 1:**
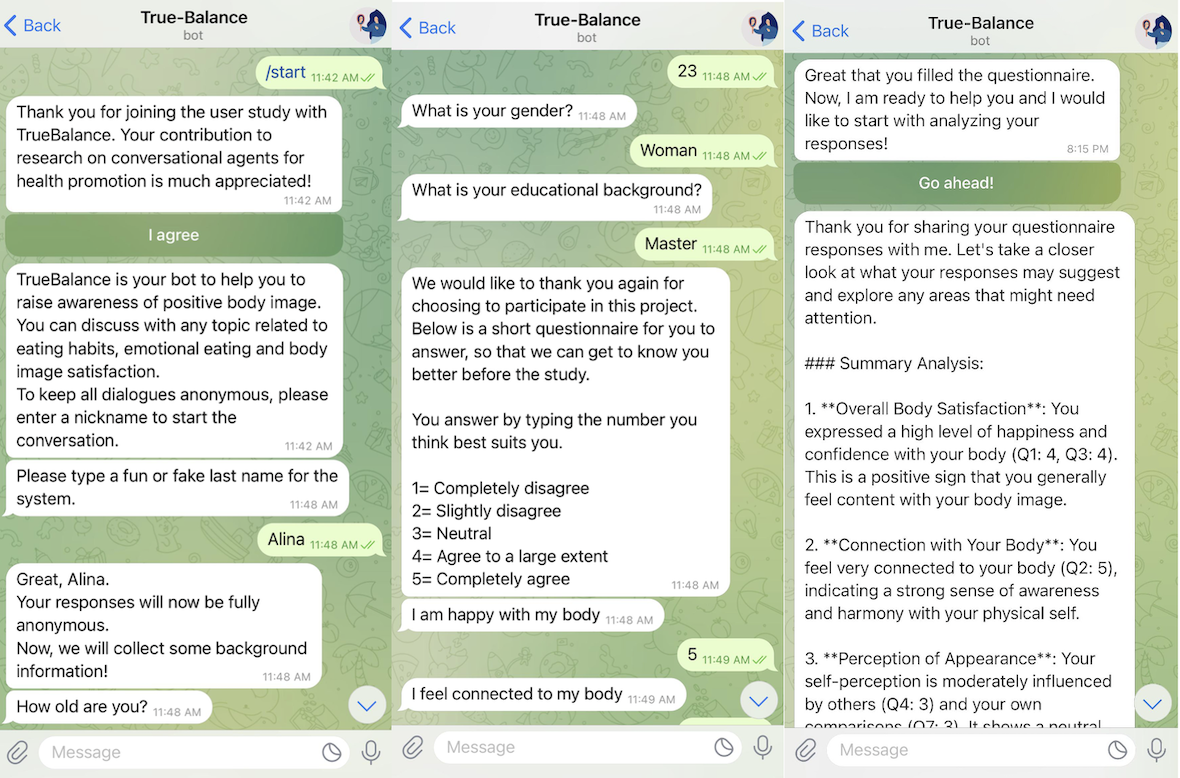
Interface of the conversational artificial intelligence agent (TrueBalance).

A refined version of TrueBalance was developed using OpenAI’s GPT-4o model to enhance conversational quality and contextual memory. GPT-4o was assessed as suitable for providing health-related guidance with minimal risk of misinformation and hallucination. The model was accessed via OpenAI’s Assistant application programming interface. To improve the personalization, an automated AI agent framework was implemented, which guided participants to answer questions from the Body Image Satisfaction Questionnaire [21] before engaging in the conversation. Each user was assigned a distinct conversation thread, which allowed TrueBalance to retain context within a given user conversation. The final implementation of TrueBalance was deployed through connecting TrueBalance to Telegram (Telegram FZ LLC; Figure 1), which was selected for its secure and anonymous communication features, allowing users to engage with the AI agent in a private, encrypted messaging environment. ManyChat (ManyChat Inc) [23], an AI agent–building platform, was used to facilitate structured conversation automation.

### Data Collection

#### Semistructured Interviews

A qualitative interview study was conducted to examine participants’ experiences with TrueBalance. Semistructured interviews were conducted before and after the interaction to explore participants’ understandings of body image and the perceived impact of conversation with the AI agent. The interview template was guided by previous literature on the multidimensional characteristics of body image that include cognitive, affective, and behavioral aspects of thoughts and feelings toward one’s body [1,14].

The preinteraction interview was conducted before the use of TrueBalance and lasted approximately 30 minutes. It focused on participants’ baseline views regarding body image, self-perceived health, emotional well-being, and prior engagement with digital technologies for health. Participants were also asked about their expectations and potential concerns related to using an AI tool for conversations regarding body image. Questions were formed in an open-ended manner, including questions such as “Are there any activities or habits that help you maintain a positive view of your body? What are they?” and “Do you think these technologies, like social media, affect your body image? If so, how have they influenced the way you feel about your body?”

Following a period of 7 days of engagement with the agent, participants took part in a second interview of similar duration. This postinterview explored their experiences of using the agent, including perceptions of usability, content relevance, and the extent to which the agent contributed to their self-reflection or body image awareness. Participants were asked questions such as “After using TrueBalance, did you notice any daily activities that make you feel satisfied with your body? If so, what kind of activities?” and “Did the agent help you manage your feelings regarding body image? Why or why not?”

All interviews were conducted online, audio-recorded with participants’ consent, and transcribed verbatim. The interview template from preinterviews and postinterviews is available as Multimedia Appendix 3.

All interviews were conducted by a researcher with a background in public health and qualitative methods to ensure consistency in data collection. Interviews were audio-recorded with participants’ consent and transcribed verbatim. Interview transcriptions were analyzed by 3 members of the research team representing different disciplinary backgrounds. Saturation of data was discussed between research team members during data collection and analysis. Data collection continued until thematic saturation was reached, with no new codes emerging during the final interviews.

#### Questionnaire

Participants’ subjective experience of their body image was assessed using the Body Image Satisfaction Questionnaire [21], administered during pre- and postinteraction phases. The questionnaire consisted of 18 items evaluating attitudes toward one’s own body and physical appearance, which was adapted from a previously validated instrument used in studies of body image in adolescents [21]. Responses were recorded on a 5-point Likert scale ranging from “completely disagree” to “completely agree.” Negatively worded items were reverse-scored so that higher scores indicated greater body image satisfaction. This measure enabled comparison of self-perceived body image before and after interaction with the TrueBalance. The Body Image Satisfaction Questionnaire [21] was used as a background variable for participants. The System Usability Scale (SUS) was administered after the intervention [24]. Participants responded on a 5-point Likert scale, covering aspects such as ease of use, interface consistency, and confidence in using the system.

### Participants

A convenience sampling strategy was used to recruit 15 participants. Participants were recruited through recruitment posters displayed at the university and an open invitation distributed through researchers’ networks. The posters included information about the study’s purpose, inclusion criteria, and contact details to ensure transparency and informed and voluntary participation. Participants were eligible if they met the following criteria: (1) aged 20-30 years, (2) in self-reported good health, (3) no current or prior diagnosis of an eating disorder, and (4) fluent in English. Individuals who did not meet these criteria were excluded.

The sample consisted of healthy young adults who were not diagnosed with body image disturbances. This limits the generalizability of our findings to clinical or high-risk populations, but the approach was intentional to explore the acceptability of such conversations. The aim was not to evaluate clinical effectiveness but to understand how users without diagnosed body image concerns might interact with such a tool in everyday settings. Since the agent was framed as a tool for preventative mental health instead of medical intervention, selecting participants from the general population provided a more feasible sample than individuals with diagnostic body image disturbances.

### Participant Characteristics

The study sample consisted of young adults (aged between 22 and 30 years) with varying levels of body image satisfaction (Table 1). Most participants were female (11/15). All participants reported having previously interacted with one or more AI agents. Most participants (10/15) reported a score of ≥60 in the Body Image Satisfaction Questionnaire before the intervention [21]. Most participants did not report significant body image concerns; however, several reflected on prior experiences, subtle insecurities, or social pressures, indicating that body image remains relevant even among generally healthy individuals.

**Table 1:** Participants’ sociodemographic characteristics (age and sex), previous experience with artificial intelligence (AI) agents, perceived body image satisfaction before and after interacting with the AI agent, and scores from the System Usability Scale.

ID	Age (years)	Sex	AI agents previously used^a^, n	Body Image Satisfaction Questionnaire score^b^ (max=90)			System Usability Scale score (max=105)
				Before	After		
P1	24	Female	3	60	61	71	
P2	23	Female	2	53	55	83	
P3	22	Female	1	79	83	89	
P4	28	Female	1	53	55	71	
P5	28	Female	2	69	63	74	
P6	23	Female	1	75	77	86	
P7	24	Female	2	61	67	88	
P8	25	Male	1	64	63	75	
P9	28	Female	1	73	74	64	
P10	30	Male	1	65	64	83	
P11	23	Male	1	47	54	83	
P12	25	Female	3	74	74	81	
P13	25	Female	1	65	71	88	
P14	28	Male	1	69	75	82	
P15	23	Female	1	65	62	63	

^a^The previous experience with the AI agent variable reflects the number of AI agent platforms with which participants reported prior interaction. These included Google Assistant, Amazon Alexa, Apple Siri, and ChatGPT.

^b^The Body Image Satisfaction Questionnaire [21] includes 18 items on a 5-point Likert scale, with negative items reverse-scored so that higher total scores indicate greater satisfaction. Scores range from 18 to 90 and are divided into 5 categories: “Extremely Satisfied,” “Very Satisfied,” “Satisfied,” “Dissatisfied,” and “Very Dissatisfied.”

### Analytical Approach

This study used a qualitative methodological approach grounded in a constructivist paradigm [25,26] to explore how young adults understand body image and the dynamics of conversations with the AI agent. This paradigm assumes that body image is not a fixed or objective entity but a socially and emotionally embedded experience shaped by language, discourse, context, and self-reflection [27,28]. A phenomenological orientation underpinned the analysis [29], emphasizing the exploration of lived experiences as expressed through participants’ narratives [30].

The primary data source included the semistructured interviews conducted before and after the interaction. The interviews’ transcripts were analyzed using thematic analysis, guided by Braun and Clarke’s [31] 6-phase approach. This approach allowed an inductive coding strategy, including a definition and refinement of themes as an iterative and active process in collaboration between the researchers. The analysis was conducted using ATLAS.ti software (Lumivero).

Thematic analysis was conducted iteratively in different stages (Multimedia Appendix 4). First, all researchers familiarized themselves with the raw data. Initial coding was performed by the first author (XZ), who reviewed all transcripts line-by-line using an inductive approach. After that, a preliminary codebook was developed and iteratively refined in consultation with SK and AZ, who also reviewed a subset of the transcripts to ensure consistency and analytical rigor. The final themes were jointly discussed and defined together by all researchers. Coding was supported by ATLAS.ti.

Text-based conversations between participants and TrueBalance were reviewed and thematically categorized according to topic domains. This descriptive content analysis was used to identify common themes across user-agent exchanges [32].

Discrepancies in interpretation were discussed with the research team until consensus was reached. Analytical memos were used throughout the process to support reflexivity. The AI-user interaction logs were analyzed in parallel to identify conversational themes and triangulate findings from the interviews. Researchers’ interdisciplinary backgrounds in public health, data science, and social sciences allowed a multifaceted approach to data analysis, which ultimately aimed to capture the nuances of AI-guided conversations, rather than proposing an AI agent as a solution to body image–related problems.

The quantitative data collected through questionnaires (Body Image Satisfaction Questionnaire [21] and SUS [24]) were analyzed descriptively. Due to the small sample size (N=15), no inferential statistical analyses were conducted. Instead, questionnaire scores were used to support and contextualize the qualitative findings.

### Ethical Considerations

This study received ethical approval from the Swedish Ethical Review Authority (reference number 2024-03938-01). All participants provided both written and verbal informed consent for participation in the study and for the publication of pseudonymized data excerpts. Prior to signing the consent form, participants were provided with detailed written and oral information regarding the study purpose and potential risks associated with TrueBalance. Participants were explicitly informed that the agent was an early-stage prototype and that its responses might occasionally include inaccurate or incomplete information. They were also made aware of their right to withdraw from the study at any time. To protect privacy and confidentiality, all participants were assigned pseudonymous identifiers, which were used in all data analysis and reporting. Upon completion of the study, participants received a small financial compensation in the form of a gift card valued at 300 SEK (US $31.50). The amount of compensation was not disclosed in the recruitment materials to avoid influencing participation.

## Results

### Maintaining Body Image Without the Conversation With an AI Agent

#### Overview

Young adults’ strategies to maintain body image without the conversation with the AI agent were divided into two main themes: (1) understanding factors influencing body image and (2) strategies for improving body positivity (Textbox 1).

**Thematic analysis of preinterviews, including main themes and subthemes of maintaining body image awareness without the conversation with an artificial intelligence agent.**

**Understanding factors influencing body image**

- Self-acceptance and confidence as an essence of body image
- Valuing body functionality
- Body image insecurities
- Influence of cultural and societal standards
- The paradoxical influence of social media
- Body image across life stages

**Strategies for improving body positivity**

- Social support networks
- Exercise and physical well-being
- Positive self-talk
- Use of makeup and cosmetic enhancements

#### Theme 1: Understanding Factors Influencing Body Image

##### Self-Acceptance and Confidence as the Essence of Body Image

Young adults described positive body image to be linked with self-acceptance and self-appreciation, emphasizing an internal sense of confidence rather than external validation. They highlighted the psychological dimension of body image, conceptualizing it as a mental state rooted in confidence and emotional well-being, rather than an objective assessment of physical appearance.

A positive body image will be related to mental factors. You’re confident with it, you look healthy. It would show on the skin and the body.

P2, 23, female

Body image resilience was characterized by emotional stability toward changes in physical appearance. Maintaining satisfaction with one’s body was seen as a protective factor against societal pressures.

I’m content with my status, and I wouldn’t be affected by my body’s changes emotionally or physically.

P3, 22, female

One participant viewed positive body image as a neutral stance toward one’s body, suggesting that an overemphasis on physical appearance could contribute to distress.

For me, it means that I have a neutral body image. If you associate feeling good about your body (...), if it changes, then that can be distressing, even if it changes in a good or bad.

P1, 24, female

##### Valuing Body Functionality

Participants described positive body image as being more about body functionality and less about physical appearance. Some expressed satisfaction with their bodies based on their ability to perform daily tasks rather than their aesthetic qualities.

I’m satisfied with how my body functions rather than how my body looks. To me, that is a good body image.

P1, 24, female

##### Body Image Insecurities

Participants described experiencing self-doubt and insecurity when evaluating their body image, particularly when comparing themselves to perceived ideals. Some expressed a lack of confidence when observing others whom they believed had more desirable body shapes.

I don’t feel confident about my body image and (feel) discouraged when seeing others who may have a better body shape or a better routine.

P3, 22, female

Others recalled specific moments of admiration toward their peers, particularly in relation to physical attributes that they felt they lacked. Height was mentioned as a factor that contributed to body image insecurity. Body image insecurities could stem from direct comparisons with others.

I kind of compared myself to her at that point. She has a very good body weight because she takes care of it so much.

P5, 28, female

##### The Paradoxical Influence of Social Media

Social media was considered significant in shaping body image awareness, often reinforcing unattainable beauty ideals. Some participants shared that exposure to curated images of fashion influencers led to subconscious comparisons and feelings of inadequacy.

People who have great aesthetics and great bodies and create skin. After looking at them, I’m like I’ll never reach that state. As a result, it’s subconscious, (...) I don’t feel so great about myself.

P4, 28, female

Despite recognizing that beauty standards distributed on social media were not realistic, some participants expressed a desire to conform to them. The role of influencers in shaping body ideals was highlighted, with concerns that portrayals of thinness as the healthiest body type could strongly impact young adults.

If the influences (in social media) start to show what could be a healthy body, I think it’s very easy to influence young adults.

P6, 23, female

Despite its negative effects, social media was also recognized as a platform for promoting body positivity and diverse representations. Some participants believed that exposure to more inclusive content could help young adults develop healthier body perceptions.

Social media will be a nice platform for spreading positivity (...). I also see a lot of content like that. It also makes me feel better as well.

P2, 23, female

##### Body Image Development Across Life Stages

Participants reflected on how their body image awareness evolved over different life stages, particularly during adolescence and young adulthood. Many reported greater detachments from external validation, fostering self-acceptance, and prioritizing opinions of close personal relationships. P8 described their early struggles with body weight and how their perspective had shifted over time.

When I was a kid, I might have felt more (societal pressure). I’m just skinny and I wanted more weight, but I could never achieve gaining weight. (...) I think now it doesn’t matter that much.

P8, 25, male

#### Theme 2: Strategies for Improving Body Positivity

##### Social Support Networks

Participants described the importance of social connections in fostering body positivity. Supportive friendships were seen to counter negative self-perceptions, with some participants turning to friends for reassurance on difficult days.

If I’m having a bad body day and I’m feeling like I’m overweight (...), I’ll text my friends.

P1, 24, female

At the same time, friendships were also viewed as spaces where body positivity could be reinforced through affirmations and mutual encouragement.

Friendships are helpful in that area. (...) Like, no, you look great. No, you’re beautiful.

P4, 28, female

Beyond verbal affirmation, engaging in physical activities with friends was a strategy mentioned by participants for improving body image. One participant described how group challenges created motivation and a sense of accomplishment.

There were times when we wanted to exercise and set challenges for each other. I think challenges are good because everyone likes a challenge.

P8, 25, male

##### Exercise and Physical Well-Being

Exercise was frequently mentioned to enhance body positivity, with participants emphasizing its role in building confidence and a sense of accomplishment rather than focusing on weight loss.

Gym is a really good choice. I think doing some jogging, running around the park is also a good way to embrace nature and feel good about yourself.

P6, 23, female

Group-based activities were seen as particularly motivating, with participants noting that collective encouragement fostered a supportive environment for personal growth.

Maybe doing sports, group activities. I think it depends on the groups. We’ll try to maybe motivate you into doing more things and becoming better.

P8, 25, male

For some, regular exercise contributed to a shift in body awareness, reinforcing confidence in their physical abilities rather than placing emphasis on aesthetics.

I started to work out more and that made me feel more confident in my body (...) I feel confident the way that I feel strong and capable in ways that I didn’t before.

P9, 28, female

##### Positive Self-Talk

Engaging in positive self-talk was another strategy participants used to counteract negative body image thoughts. Some described actively reminding themselves of their worth beyond physical appearance, highlighting the role of self-compassion in reducing self-criticism.

I’ve also learned to tune out those thoughts and remind myself: you’re healthy, you’re good, you take care of yourself, and you have nothing to worry about.

P1, 24, female

##### Use of Makeup and Cosmetic Enhancements

Some participants described using makeup and fashion to enhance their appearance to feel more confident.

I still like to make up or dress myself to make my appearance better.

P7, 24, female

### Conversations With the AI Agent

The conversations between participants and the AI agent, TrueBalance, were grouped into four overarching domains: (1) body image perception, (2) body image–related eating and behavioral regulation, (3) body-focused mindfulness, and (4) social conversation with the agent. An additional default category, technical issues, captured brief mentions of service-related problems (Table 2).

Across all domains, 933 messages were exchanged by 15 participants. Each domain comprised several subcategories, illustrating how participants navigated diverse and sometimes overlapping concerns in their dialogue with the agent.

**Table 2:** Content analysis of conversations with the artificial intelligence agent, including categories, subcategories, number of messages, and number of users.

Category of the conversation and subcategory of the conversation		Users, n (%)		Messages, n (%)
**Body image** **awareness**				291 (31)
	Self-acceptance and confidence	13 (87)	128 (14)	
	Self-disclosure	9 (60)	75 (8)	
	Understanding of body image	5 (33)	27 (3)	
	Influence of cultural and societal standards	6 (40)	20 (2)	
	Skin care	5 (33)	16 (2)	
	Feedback on questionnaire	15 (100)	15 (2)	
	Social media influence	2 (13)	10 (1)	
**Body image–related eating and behavioral regulation**				423 (46)
	Diet plan	9 (60)	175 (19)	
	Biomedical determinants of eating disorder	2 (13)	96 (10)	
	Weight management	6 (40)	60 (6)	
	Exercise plan	7 (46)	43 (5)	
	CBT^a^ technique	7 (46)	20 (2)	
	Emotional eating	2 (13)	12 (1)	
	Calorie count	1 (6)	9 (0.9)	
	Sleep problems	2 (13)	8 (0.8)	
**Body-focused mindfulness**				109 (12)
	Practicing gratitude	7 (46)	49 (5)	
	Meditation	4 (26)	34 (4)	
	Mindful eating	3 (20)	19 (2)	
	Journaling	1 (6)	7 (0.7)	
**Social conversation with agent**				106 (11)
	Expressions of appreciation	7 (46)	57 (6)	
	Exploration of agent’s function	6 (40)	24 (2)	
	Conversational politeness	6 (40)	22 (3)	
	Request for optimizing format	2 (13)	3 (0.3)	
**Technical errors**				6 (0.6)
	Out of service	4 (26)	6 (0.06)	

^a^CBT: cognitive behavioral therapy.

Body image awareness constituted one-third of the messages (299/933, 32%). Conversations were centered on the psychological and social aspects of how participants viewed themselves. Discussions on self-acceptance and confidence appeared most frequently, reflecting a shared interest in cultivating emotional resilience and a compassionate relationship with the body. Other subtopics, such as understanding of body image, influence of cultural and societal standards, skin care, and social media influence, highlight how participants grappled with external and internalized ideals. Less frequently, participants mentioned topics such as practicing gratitude and sleep problems, often within personal or reflective contexts.

In body image–related eating and behavioral regulation (423/933, 46% messages), participants engaged with the agent on food practices, health behaviors, and concerns about disordered eating. Conversations about diet plans were particularly prominent, suggesting a strong interest in structured approaches to eating. Other themes included eating disorders, weight management, and exercise plans, often discussed in relation to personal goals or challenges. Some participants also touched on calorie counting, emotional eating, and cosmetic enhancements, indicating the complexity of how body-related behaviors are experienced and regulated.

The domain of body-focused mindfulness (109/933, 12% messages) featured interactions where participants turned to the agent for strategies related to emotional well-being and mental regulation. Discussions around practicing gratitude and meditation were common, with participants using the agent to support mindfulness routines or coping mechanisms. Other topics like mindful eating and journaling appeared as ways to cultivate a more grounded and accepting awareness of the body.

Finally, in social conversations with the agent (106/933, 11% messages), participants explored the agent’s role as a conversational partner. These exchanges included expressions of appreciation and curiosity about the agent’s function. Some commented on the politeness of the agent or offered feedback on its format, suggesting that the relational tone of the interaction played a meaningful role in the user experience.

A small number of interactions were categorized under technical issues, including brief instances where participants noted that the system was not responsive or functioning as expected.

### Advantages and Drawbacks of Using an AI Agent for Body Image Awareness

#### Overview

The thematic analysis of postinterviews identified three themes in perceived advantages and drawbacks of conversations with the agent: (1) facilitating body image awareness and self-reflection, (2) availability of conversational support, and (3) discontinuities in user engagement (Textbox 2).

**Thematic analysis of postinterviews, including main themes and subthemes of advantages and drawbacks of using an artificial intelligence (AI) agent for body image awareness.**

**Facilitating body image awareness and self-reflection**

- Increased understanding of eating disorders and emotional eating
- Facilitating reflective thinking
- Promoting self-acceptance and emotional resilience
- Reaffirmation of preknowledge
- Reflecting on social media’s role
- Recognition of the importance of early awareness

**Availability of conversational support**

- 24/7 online availability and rapid responsiveness
- Private and nonjudgmental communication
- Anthropomorphic connection: emotional support, proactivity, and personalization
- Integrating AI agent into daily life

**Discontinuities in the user engagement**

- Desire for continuing conversations declined over time
- Issues with agent responsiveness
- Overwhelming message
- Challenges in translating health intentions into actions

#### Theme 1: Facilitating Body Image Awareness and Self-Reflection

##### Increased Understanding of Eating Disorders and Emotional Eating

Participants highlighted that the conversation with the agent enhanced their knowledge of eating disorders and emotional eating. Most participants reported that they had gained insights into balanced eating habits and the psychological factors influencing disordered eating behaviors, following a conversation with the agent. The accessibility and structured nature of the AI agent were viewed as valuable in acquiring dietary knowledge.

It was good to know what is healthy, and I would learn a couple of facts about how to eat more balanced, especially when I’m not feeling hungry or if I’m feeling depressed.

P1, 24, female

For some participants, the agent served as a means of affirming existing knowledge, instead of changing their body image. Some expressed that they already held positive beliefs about body image, but receiving external confirmation reinforced their confidence.

I don’t think my opinion (of body image) changed that much, but I got affirmation of that. I was thinking like I was on the right path.

P9, 28, female

##### Facilitating Reflective Thinking

The agent also encouraged self-reflection, leading participants to critically assess their body image. Some described an increased self-kindness and cultivating positive thought patterns.

It expanded my knowledge that body positivity was more of self-acceptance and an appreciation for all body types, regardless of what society may make us think.

P11, 23, male

##### Promoting Self-Acceptance and Emotional Resilience

Many participants expressed that the agent provided a supportive environment for self-acceptance and emotional resilience. This helped them to challenge self-critical thoughts and adopt a more positive self-view.

I should be kinder to myself, and sometimes I should take small steps to build a habit, a pattern of thinking, having positive thinking, and having a nice relationship with myself.

P2, 23, female

#### Theme 2: Availability of Conversational Support

##### 24/7 Online Availability and Rapid Responsiveness

The agent’s accessibility was recognized as a key advantage, particularly when traditional support systems, such as friends or health care professionals, were unavailable. The ability to receive instant feedback provided reassurance.

(The agent) can give instant support. If I have a thought right now, my doctor might not be available right now to give me some information on what I need or make me calm down.

P12, 25, female

Some participants reported interacting with the agent during late hours, finding comfort in its responsiveness in contrast to human support, which often involves delays.

I communicated with it at midnight. I tend to eat some snacks, some chocolates, and ice cream. That’s always seen as unhealthy, right? I send it because, at that time, all my friends are sleeping—only TrueBalance answers me.

P13, 25, female

##### Private and Nonjudgmental Communication

Privacy and nonjudgmental communication were frequently mentioned as advantages of using the agent. Participants expressed feeling more comfortable discussing sensitive topics with the AI agent compared to human interactions. They noted that the agent provided a space where they could speak openly without fear of judgment. This increased sense of comfort was frequently mentioned.

If I am talking to a human, I probably would be more conservative. Some questions I wouldn’t even ask, especially if it’s someone I’m not familiar with. But with (an agent) (...) I feel so comfortable telling everything to it.

P15, 23, female

##### Emotion-Sensitive Conversations

Participants appreciated the agent’s emotional support and sensitivity in phrasing responses in a caring way. Some engaged with the agent in a more personal manner and included emotional expressions in their responses. This resulted in a perception of the agent as empathetic and validating.

If I asked specific questions and included how I felt that day, it would show empathy, which was nice. I didn’t expect that. It would try to make you feel like, okay, your feelings are valid.

P4, 28, female

The agent’s perceived warmth and ability to convey a sense of care were also noted, reinforcing a perception of supportiveness. Some participants appreciated the agent’s ability to guide conversations based on previous dialogue rather than simply providing answers.

In the beginning, I really liked the fact that it gave you some prompt questions—things I could discuss. It opened meaningful conversations that could move forward depending on my responses.

P5, 28, female

##### Integrating Conversational Advice Into Daily Life

Participants believed that the agent helped them support decision-making in relation to nutrition and self-care. This served as a motivation to implement new habits into daily life.

I was looking into how to implement habits in my daily life. I think that can be helpful—if I want to go to the gym regularly, but not excessively.

P14, 28, male

The agent helped participants to shift their perspectives and find reassurance, and thus process emotions related to body image. P2 (23 years of age, female) stated that “asking the AI agent made me feel better and opened my mind to the thoughts I had.” Similarly, P12 mentioned that the agent gave reassurance for handling feelings:

It helped by giving reassurance on how to handle my feelings or other people’s feelings.

P12, 25, female

#### Theme 3: Discontinuities in the User Engagement

##### Lack of Engagement With the Agent Over Time

Participants noted that their engagement with the agent declined over time, often due to difficulties in sustaining meaningful conversations. Some described struggling to come up with new topics to discuss. Others expressed uncertainty about how to initiate new conversations after initial topics:

I struggled with how to prompt the conversation. (...) We would discuss something, and then we would end the conversation, and then there was nothing more to discuss. I wouldn’t know how to start again.

P5, 28, female

##### Issues With the Agent’s Responsiveness

A common concern was the agent’s tendency to generate repetitive responses, which some found redundant. Sometimes the agent failed to recognize the user’s input, either by ignoring follow-up queries or restarting conversations.

Maybe because the topics were interrelated, some of the responses were a bit repetitive. That’s probably why, at times, it felt a bit repetitive.

P4, 28, female

P5 and P8 described moments when the agent stopped responding entirely. P5 mentioned asking a question to the agent, but not receiving a reply*.* Additionally, a few participants mentioned frustration with having to repeat basic information.

##### Overwhelming Messages

While some participants appreciated detailed responses, others found the agent’s messages too lengthy. P8 and P13 mentioned that the amount of text provided felt excessive, leading to disengagement.

When I asked about emotional eating, it provided a long text. For me, it makes me feel anxious because I just asked a simple question, and the feedback was a flood of information. Sometimes I find myself lost in the long text.

P13, 25, female

##### Challenges in Translating Health Intentions Into Actions

Participants admitted that they struggled to translate the agent’s suggestions into actionable steps, often citing a lack of motivation after the immediate impact.

Some people might be looking for an emotional or motivational boost, which (the agent) doesn’t provide as well as a video or an inspiring speech.

P8, 25, male

## Discussion

### Principal Results

This study investigated the benefits and drawbacks of using an LLM-based conversational agent aimed at raising awareness of body image among young adults through providing conversational support. As a response to RQ1, the study shows that young adults’ body image awareness was connected to self-acceptance, confidence, and valuing body functionality. Participants used several strategies for raising awareness on body image without the conversation with the AI agent, ranging from social support networks to exercise and positive self-talk. As a response to RQ2, the conversations with the AI agent were categorized into (1) body image awareness, (2) body image–related eating and behavioral regulation, (3) body-focused mindfulness, and (4) social conversation with the agent.

In response to RQ3, the study identified three themes in perceived advantages and drawbacks of conversations with the agent: (1) facilitating body image awareness and self-reflection, (2) availability of conversational support, and (3) discontinuities in user engagement. Participants appreciated the agents’ ability to facilitate knowledge acquisition and information on body image–related topics. The instant availability provided immediate support, making it a tool for individuals seeking quick responses to their concerns. Its private and stigma-free nature allowed participants to engage openly with sensitive topics without fear of judgment. However, areas for improvement were identified regarding conversational adaptability, reduction of response repetition, and interactivity to sustain long-term engagement.

### Comparison With Prior Work

TrueBalance differs from existing chatbots such as Tessa [33], KIT, and Topity [12,13] in accessibility, methodology, and intervention design. Tessa and TrueBalance are integrated into popular social media platforms, reducing accessibility barriers. In terms of methodology, KIT relies on a structured decision tree developed by Proxima using Iris Conversational Intelligence, while Topity uses a gamified, avatar-based approach. Tessa provides limited technical detail, whereas TrueBalance uses AI to deliver adaptive, context-aware interactions. This dynamic design enhances personalization and flexibility but may also present risks related to user safety.

These chatbots also differ in assessment features, target demographics, and duration of engagement. Only Tessa and TrueBalance include parametric assessments to support structured self-reflection. KIT offers supportive conversation without self-measure tools, and Topity provides microinterventions without tracking user progress. Tessa targets adolescents (aged 13-18 years) for 72-hour engagements, while TrueBalance focuses on young adults over approximately 7 days, which is longer than KIT’s reported use.

In this study, participants did not report any harmful consequences from using TrueBalance, reinforcing its potential as a safe and supportive tool for body image discussions. However, we did not specifically assess the information provided by the AI agent from the perspective of professional psychiatrists or dietitians. Ensuring that TrueBalance remains both beneficial and nontriggering is a crucial consideration for future implementations, particularly in refining its content to align with expert recommendations.

### Recommendation for Future Design

Future development must prioritize personalization in 3 interrelated dimensions, that is, user-level personalization, system-level limitations, and institutional-level ethical safeguards [34-36]. While personalization enhances engagement and perceived empathy, it also introduces risks of misunderstanding and cognitive overload. Balancing these competing demands remains a central design challenge.

Personalization should extend beyond surface tailoring to include anthropomorphic design, adaptive notifications, context-aware prompts, and access pathways to credible health information and professional support. This study shows that anthropomorphic design must be weighed against the risk of agent responsiveness issues, where delayed or generic replies can undermine perceived reliability. Anthropomorphic features, such as emotional responsiveness and adaptive tone, can strengthen relational trust; yet, their misuse or inconsistency may create unrealistic expectations or diminish credibility. Excessively long or overwhelming messages may also reduce usability, suggesting that information should be carefully paced and structured to avoid cognitive overload. Importantly, AI agents should function as a bridge rather than a replacement for professional care, facilitating expert input when users’ needs exceed self-help capacities. Anthropomorphic features, such as emotional responsiveness and adaptive tone, can strengthen relational trust, yet their misuse or inconsistency may create unrealistic expectations or diminish credibility [37].

Our findings further revealed that participants often struggled to translate the awareness gained from conversations with the agent into sustained behavioral change. While users reported increased understanding of emotional eating, body awareness, and self-acceptance, many described difficulties integrating these insights into everyday practices. This gap between cognitive reflection and behavioral implementation reflects a broader limitation of digital self-care tools, where momentary motivation may not lead to long-term change. To address this, future conversational agents can integrate behavior-support mechanisms, such as personalized goal setting and feedback loops that help users operationalize insights into actionable steps while maintaining autonomy and emotional safety [38].

System-level limitations in responsiveness emerged as a consistent concern among participants. These findings suggest that conversational responsiveness and coherence are key determinants of perceived feasibility in AI agents. When the dialogue lacked contextual awareness or failed to build upon previous input, users interpreted the agent as technically limited or emotionally detached. From a design perspective, these challenges highlight the need for adaptive dialogue management and context-preserving system architectures. Moreover, implementing feedback-driven adjustments may help prevent repetition. Maintaining stable responsiveness and contextual sensitivity plays an important role in enhancing the functional reliability and perceived trustworthiness of conversational AI agents [38].

### Limitations

The study has limitations that should be considered when interpreting the results. The sample consisted predominantly of highly educated individuals, which may limit the generalizability of the findings. Education level has been linked to health literacy and digital engagement, potentially influencing participants’ ability to interact effectively with the AI agent [7,39]. Future research should aim to include individuals from a wider range of educational backgrounds to determine whether agent-based interventions are equally effective across varying literacy levels. Additionally, the study did not extensively explore differences in agent engagement across various languages. Since body image concerns are influenced by cultural norms and social expectations [25,40], it is essential to evaluate the effectiveness of AI-driven strategies for individuals from diverse linguistic and cultural backgrounds.

A critical limitation of this study is that we have not conducted a systematic evaluation of the accuracy of the agent’s responses [41]. This presents potential ethical risks, particularly in its deployment among young adults with a history of eating disorders [34]. Given the sensitivity of body image concerns and the potential vulnerability of users, inaccurate, misleading, or overly generalized responses could inadvertently reinforce harmful thoughts or behaviors. For that reason, this study was conducted among healthy young adults with relatively high educational backgrounds. The absence of expert validation in assessing the AI agent’s content further underscores the need for rigorous quality control and continuous monitoring to ensure that the intervention remains safe, supportive, and evidence-based [42]. Future research should investigate the use of the LLM-based agent with another sample consisting of individuals seeking support for body image–related questions, such as participants with diagnosed eating disorders or elevated body dissatisfaction.

### Conclusions

This study demonstrated the acceptability of an LLM-based AI agent in fostering body image awareness among young adults. The agent’s ability to provide instant and emotionally supportive interactions demonstrates the potential to use the agent in preventative health. Challenges remain in sustaining long-term user engagement, which highlight the need for multidimensional personalization of the agent.

## Appendix

Multimedia Appendix 1 Knowledge base.

Multimedia Appendix 2 TrueBalance instructions.

Multimedia Appendix 3 Interview template.

Multimedia Appendix 4 Thematic analysis.

## Abbreviations

AI: artificial intelligence
CBT: cognitive behavioral therapy
LLM: large language model
RQ: research question
SUS: System Usability Scale

## Data Availability

Datasets generated or analyzed during this study are available from the corresponding author on reasonable request.
