# Supplementary material for: Exploring Body Image Awareness With a Large Language Model–Based Conversational Agent: Qualitative Study With Young Adults
Source: J Med Internet Res. 2025 Nov 17;27:e78829. doi: 10.2196/78829 (PMC12670058; doi:10.2196/78829)
Supplement: Multimedia Appendix 1 [file jmir_v27i1e78829_app1.pdf]

Eating Disorders from a Biomedical Perspective

EDs involve long-lasting disturbances in eating behaviors that negatively affect both health and psychosocial well-being \cite{apa2013dsm}. While concerns about health, weight, or physical appearance are occasional for many, some individuals may develop a fixation or intense focus on losing weight, managing body size or shape, and strictly regulating their dietary intake. Such behaviors might indicate an ED \cite{nimh2021eating}. EDs are diagnosed according to specific criteria outlined in major diagnostic manuals, such as the Diagnostic and Statistical Manual of Mental Disorders (DSM-5) and the International Classification of Diseases (ICD-10) \cite{mari-sanchis2022eating}. The range of these psychiatric disorders encompasses conditions like Anorexia Nervosa (AN), Bulimia Nervosa (BN), Binge Eating Disorders (BED), and Avoidant/Restrictive food intake Disorder (ARFID) \cite{nimh2021eating}.

AN is characterized by a critically low body weight and body mass index (BMI), driven by an intense fear of weight gain and self-restrictive nutrition practices. Those who suffer from it may resort to excessive physical activity and purging to control their weight \cite{gorgas2024eating}. Of all psychiatric illnesses, AN has the most severe mortality rate, underscoring the urgency for effective treatment and management \cite{neale2020anorexia}. DSM-5 classifies different severity ratings for AN based on BMI measurements: the most critical level is indicated by a BMI less than 15 kg/m^2, followed by serious, moderate, and mild categories, with respective BMI ranges of 15-15.99 kg/m^2, 16-16.99 kg/m^2, and a BMI of 17 kg/m^2 or higher for the mildest classification \cite{zipfel2015anorexia}.

Individuals suffering from BN experience consistent cycles of consuming abnormally large quantities of food, coupled with a sense of lost control during these binges. Such episodes are consistently compensated by efforts to balance the effects of overeating, which include deliberate vomiting, misuse of laxatives or diuretics, fasting, or intense physical exercise \cite{nimh2021eating}. In contrast, those with BED also experience similar uncontrolled, recurrent eating episodes but do not engage in compensatory behaviors, which often leads to being overweight or obese. The fourth category, ARFID, is characterized by a significant avoidance or limitation of food consumption. It differs from other EDs as it does not always involve a considerable focus on body image or fear of weight gain \cite{nimh_eating_disorders}.

Primarily affecting young individuals, these EDs significantly damage mental and physical health. They most commonly emerge during late adolescence and early adulthood, leading to disruptions in educational and professional efforts for those affected \cite{herpertz_diagnosis_2011}. Up to 17.9\% of adolescent girls are impacted by EDs \cite{silén2022worldwide}, whether in whole or partial forms, posing significant risks to their health and well-being. Although clinical interventions can help, they often come with high costs and may not be widely accessible. Therefore, considering the widespread occurrence of these disorders, there's a clear need for preventive measures \cite{mcknight2003risk}.

The investigation into the risk factors for developing EDs reveals a complex interplay between environmental, social, physiological, and biological determinants. Focusing on the biological aspects, comprehending how genetic predispositions and physiological factors influence an individual's susceptibility to conditions like AN, BN, and BED is required.

Knowledge of biological risk factors can guide the development of specialized, research-based intervention programs, enabling healthcare providers to address the underlying physical components of these disorders, leading to more individualized and potentially more effective treatment outcomes \cite{breithaupt2018updates}. The forthcoming sections delve into these biological factors: how genetic composition, neurobiological mechanisms, and other inherent physiological conditions contribute to the onset of EDs. This analysis is based on insights from studies examining the complex origins of these conditions, providing a clearer picture of the internal risk factors predisposing individuals to such challenging and life-altering disorders.

Genetic Factors

The examination of genetics in predisposing individuals to EDs through advanced genomic studies and research techniques highlights a significant hereditary component. Family and twin studies have been pivotal in this context, particularly revealing that monozygotic twins show higher concordance rates for eating disorders than dizygotic twins \cite{thornton2011heritability}.

The higher concordance rates for EDs among monozygotic twins compared to dizygotic twins stem from their genetic makeup. Monozygotic twins share their entire genome, meaning they have identical genetic material \cite{bruder2008phenotypically}. This genetic similarity results in a higher likelihood that both individuals in a monozygotic twin pair will develop an ED if it has a genetic component. In contrast, dizygotic twins share approximately 50\% of their genetic variation. Therefore, the difference in concordance rates between these two types of twins provides strong evidence of genetic influence in EDs. Furthermore, heritability estimates for conditions like AN, BN, and BED typically range between 40\% to 65\%, indicating a significant genetic contribution to these conditions \cite{yilmaz2015genetics}.

Genome-wide association studies (GWAS) further this understanding by identifying genetic variants that gradually increase the risk of EDs. GWAS systematically scans the genome to identify genetic variations, typically single-nucleotide polymorphisms (SNPs), associated with a specific disease. In a study published in the American Journal of Psychiatry \cite{duncan2017significant}, GWAS was performed to investigate AN's genetic underpinnings. It included 16,992 cases of individuals diagnosed with AN and 55,525 control subjects without the disorder. This research revealed a significant genetic locus for AN on chromosome 12 at location 12q13.2. This locus is in a region that overlaps six genes (IKZF4, RPS26, ERBB3, PA2G4, RPL41, and ZC3H10) and is near six additional genes (ESYT1, SUOX, RAB5B, CDK2, PMEL, and DGKA). These genes collectively contribute to various biological processes, including cell growth, immune response, protein synthesis, and metabolic functions. The most notable SNP found was rs4622308 in the 12q13.2 region. This SNP emerged as the most significant genetic variation in that area, more prevalent in individuals with AN compared with the control group. Accordingly, the GWAS results do not imply the exclusive presence or absence of genetic loci in either group but rather differences in frequency or form.

GWAS highlights the polygenic nature of EDs, showing they result from the combined effects of multiple genes, each contributing marginally to the risk \cite{abdulkadir2022eating}. The

interplay between the genetic and environmental aspects is crucial in the phenotypic expression of disorders like AN. Techniques like gene-wise analysis quantify genetic susceptibility and clarify the complex interactions involved, showing that some genetic variations may have a noteworthy impact on the phenotype of EDs. Even if discovering specific genetic loci enhances understanding of these disorders' complex genetics, it does not definitively predict the development of disorders like AN \cite{duncan2017significant}. This underlines the importance of a comprehensive approach, integrating genetic variations with environmental factors, in developing treatments and advancing precision medicine. The discoveries from GWAS often involve variants with relatively small effects on disease risk, requiring further research to understand their functional implications.

\textit{INCLUDE OTHER GENETIC STUDIES}

Neurobiological Aspects

Recent neuroscience advancements have revealed the intricate neurobiological foundations contributing to EDs. These recent developments have clarified the functions associated with altered brain structure, neurotransmitter systems, and neuroendocrine functions, all of which collectively impact the development and maintenance of EDs \cite{frank2019neurobiology}. Simultaneously, the complex relationship between psychosocial factors and neurobiological anomalies presents challenges in advancing neuroscientifically based treatments for EDs, indicating the need for extended research strategies to understand and address these disorders effectively \cite{kaye2013nothing}.

In pursuit of this objective, neuroimaging techniques, including magnetic resonance imaging (MRI), functional magnetic resonance imaging (fMRI), and positron emission tomography (PET), have played a pivotal role in investigating brain structures and functions related to EDs \cite{frank2019neurobiology}. These imaging methods have provided helpful insights into the neurobiological underpinnings of EDs, offering a thorough comprehension of the structural and functional distinctions within the brain implicated in these pathologies.

MRI provides high-resolution anatomical brain images, allowing researchers to examine structural abnormalities or differences in various brain regions. A study published in CSN Spectrum \cite{frank2015advances} revealed that specific alterations in brain gray matter volume were observed in adults and adolescents with AN during illness and post-recovery and in adults with BN. The gray matter, or cerebral cortex, is the brain's outer surface composed of densely concentrated nerve cells. The cerebral cortex is subdivided into the frontal, temporal, parietal, and occipital lobes, each entailing various functions. MRI made it feasible to ascertain an increase in the left medial orbitofrontal gyrus rectus volume, a region critical in controlling food intake, and in the right insula, which processes taste and interoception. The study's authors claim that an altered function within the orbitofrontal cortex may contribute to self-starvation and that the altered insula function may be related to the persistent precipitation of being overweight, two of the main symptoms of AN. Importantly, studies have indicated that altered cortical volumes in individuals with AN are related to the severity of the illness. As individuals undergo weight restoration, these cortical changes tend to normalize and return to normalcy during long-term recovery in adults \cite{wagner2006}.

Insights obtained through MRI can be further enhanced fMRI, which enables the assessment of cerebral activity in response to various tasks or stimuli related to eating behaviors \cite{frank2019neurobiology}. By measuring changes in blood flow, fMRI can identify brain regions that are activated or deactivated in response to food cues, self-control tasks, or emotional triggers in individuals with EDs. This helps elucidate the neural mechanisms underlying abnormal eating patterns and impulse control.

For individuals suffering from BN, similar orbitofrontal cortex changes were observed alongside alterations in the dorsal striatum \cite{frank2015advances}. The dorsal striatum serves as a neural hub, processing information from our senses, motivations, and physical condition. Its primary function is facilitating appropriate actions to achieve favorable outcomes, such as rewards, while avoiding unfavorable ones \cite{surmeier2010}.

This insight was gained by examining dopamine-related brain activity and taste-reward condition tasks. It suggests that the reward circuitry appears less responsive in the cases of BN and obesity, in contrast to the heightened sensitivity observed in AN \cite{frank2015advances}. These observations imply that the human brain can adapt its reward system when exposed to extreme food intake patterns. These adaptations may, in turn, pose challenges for individuals in their efforts to return to normal eating behaviors.

The relationship between the reward system and eating disorders is a subject of significant interest in the field of neuroscience. The reward system, a complex network of neural circuits involving neurotransmitters such as dopamine, plays a pivotal role in regulating our responses to pleasurable stimuli, including food. This system is integral to our survival, as it motivates behaviors essential for obtaining nutrition and other essential rewards \cite{frank2013altered}. In this context, PET emerges as a valuable imaging technique for investigating potential alterations in neurotransmitter activity among individuals with EDs, potentially contributing to their behavioral and symptomatic expressions. PET allows for observing neurotransmitter receptor distribution and density within various brain regions. It involves using specialized radiolabeled molecules, known as tracers, tailored to the specific neurotransmitter systems being studied. These tracers bind to neurotransmitter receptors, and the PET scanner detects emitted positrons resulting from radiolabel decay, generating precise images that clarify receptor distribution and density \cite{gulyas2008}.

The neurotransmitters that have received the most significant research attention in the context of EDs are dopamine and serotonin (or 5-hydroxytryptamine, 5-HT) \cite{diGianni2020eating}. These are often described as two of the four “feel-good” hormones in simplified contexts. The heightened interest in dopamine stems from its broad distribution of receptors within brain regions and neural pathways associated with food craving, executive function, decision-making, and impulsivity \cite{yu2022literature}. There are five different kinds of dopamine receptors: D1, D2, D3, D4, and D5. Regarding eating behaviors, the dopamine receptor D2 is particularly interesting due to its confirmed association with food-anticipatory behavior, food restriction, reward processing, and motivation \cite{peng2016dopamine}. A PET imaging study on dopamine and D2 receptors in BN has identified neurobiological patterns similar to those observed in addictive disorders, indicating dopaminergic abnormalities resembling substance abuse \cite{broft2012striatal}. This research, utilizing [11C]raclopride (a radiotracer that binds to D2 receptors) and methylphenidate (a medication that elevates dopamine levels by inhibiting dopamine

reabsorption, resulting in higher synaptic dopamine concentrations), highlights reduced D2 receptor availability and a correlation between decreased dopamine release in the striatum and the frequency of binge eating episodes. Such findings imply that BN may entail a dysregulation of the dopamine system, disrupting the brain's reward mechanism. This disruption alters how rewards are perceived, attenuating the pleasure derived from everyday activities, including eating. Consequently, individuals with BN may resort to binge eating or other compulsive behaviors in an attempt to stimulate the reward system, leading to a cycle of psychological and physical distress as they seek to self-medicate or alleviate negative emotions through food.

Similar studies have been conducted on individuals with AN. For instance, an article published in Biological Psychiatry utilized the same radiotracer, [11C]raclopride, to perform PET imaging on ten women who had recovered from restrictive-type AN, along with 12 healthy controls, to investigate dopamine dysregulation \cite{frank2005increased}. The results indicated a notable increase in [11C]raclopride binding potential within the ventral striatum among women who had recovered from AN, in contrast to the control group. Moreover, a positive correlation was observed between self-reported harm avoidance and [11C]raclopride binding in the dorsal caudate. These findings suggest that AN may be characterized by reduced dopamine levels or an increased affinity/density of D2/D3 receptors. Although these findings suggest that dopamine dysregulation could be a trait-associated element contributing to AN, establishing causality is not feasible. Instead, dopamine dysregulation may have ensued after the onset of AN \cite{johnson1990d1}.

On the other hand, it has also been observed in several animal studies that dopamine levels increase in response to rewards, including appealing food, in food-restricted animals \cite{carr2002augmentation}. This elevation in dopamine is particularly significant, considering that food restriction is a fundamental characteristic of AN \cite{gorgas2024eating}. Even though animal studies cannot fully replicate the complexities of human behavior, they provide valuable understandings of the biological mechanisms driving dietary restraint in AN.

Animal research findings indicate that dopamine sensitization correlates with food restriction, a conclusion that aligns with findings from human studies. For instance, a study conducted by Cowdrey et al. \cite{cowdrey2011increased} used fMRI to examine brain responses to food stimuli in 15 women who had recovered from AN and 16 healthy women. They found that those who had recovered from AN showed heightened activity in the ventral striatum when exposed to tasty food and increased activity in the occipital and prefrontal cortex when viewing appealing food. This is consistent with the previously described findings indicating higher dopamine activity in brain areas associated with reward among individuals with AN \cite{frank2015advances}. Additionally, women who had recovered from AN showed heightened activity in the insula and putamen when tasting non-appealing food and increased activity in the anterior cingulate cortex when observing it, suggesting a stronger desire for all types of food \cite{cowdrey2011increased}.

This aligns with the idea that individuals with AN may have an intense craving for food, consistent with the sensitization theory. The sensitization theory proposes that behavioral addictions and psychopathologies involve an excessive desire for rewards triggered by stimuli, without a corresponding increase in the pleasure derived from consuming those

rewards. This phenomenon is linked to lasting changes in dopamine-related motivation systems, termed “neural sensitization” \cite{berridge2016liking}.

The theory further suggests that robust neural systems, including mesolimbic dopamine, drive the desire for rewards. In contrast, the pleasure experienced from reward consumption is regulated by smaller and more sensitive neural systems independent of dopamine \cite{berridge2016liking}. Although dopamine itself is not examined directly by fMRI, the findings of activity in regions of the mesolimbic pathway indicate dopamine transmission. Cowdrey et al.'s findings also support previous research showing that individuals with AN pay more attention to food, meaning they may have strengthened control over their food choices. Therefore, it is likely that increased control over food intake, driven by excessive brain activity, contributes to the restrictive eating patterns seen in AN \cite{cowdrey2011increased}.

In simplified terms, dopamine is the primary neurotransmitter in the brain's reward system, whereas serotonin is an intermediary of sensations associated with satisfaction and internal solidity. The role of seratonin, 5-HT, in regulating eating behavior is firmly established. Changes resulting in decreased 5-HT activity provoke compulsive or binge eating, while those that increase 5-HT neurotransmission lead to reduced eating behavior in animals and humans \cite{blundell1986serotonin}.

Initial observations in women with active AN seem to oppose the expected pattern, showing reduced 5-HT activity. This is indicated by several factors, including decreased affinity of serotonin uptake inhibitors \cite{weizman1986high}, weakened reactions of specific hormones to serotonin-related substances \cite{monteleone1998prolactin}, lower levels of serotonin metabolites in the cerebrospinal fluid \cite{kaye1984differences}, and reduced activity of an enzyme involved in serotonin metabolism \cite{diaz2000study}. However, a study by Kaye et al.\cite{kaye1984differences} found that individuals who have recovered from AN show normal prolactin responses to agents that release 5-HT after achieving a healthy weight. This suggests a potential improvement of serotonin-related abnormalities associated with their condition. Additionally, Kaye et al. documented that levels of cerebrospinal fluid 5-hydroxyindoleacetic acid (5-HIAA) in types of AN characterized by binge eating are lower than those in restrictive forms, associating bulimic symptoms with an expected decrease in 5-HT activity.

Further research conducted by Kaye et al. \cite{Kaye1991} suggests that individuals who have fully recovered from AN show elevated levels of 5-HIAA and increased binding of 5-HT1A receptors. These findings, measured by PET, imply a primary state of heightened 5-HT activity in AN. This state may not be apparent during active disease due to reduced 5-HT activity caused by undernourishment. It is suggested that observations made during dynamic stages of the disease may distort the presence of an underlying hyperserotonergic trait, meaning that fluctuations in serotonin levels during active phases of the illness could obscure or misrepresent the true extent of serotonin dysregulation inherent in the disorder. Recent PET studies on women who have fully recovered from AN and have attained a healthy weight also support a "5-HT overactivity hypothesis", indicating reduced binding of 5-HT2A receptors in specific brain regions, which may reflect compensatory mechanisms in response to increased extracellular levels of 5-HT \cite{Kaye1991}.

Findings from studies of patients with active BN are more consistent with the theory positing that alterations leading to decreased serotonin (5-HT) activity provoke compulsive or binge eating. These findings associate the disorder with reduced synthesis, release, and neurotransmission of 5-HT, thereby highlighting the 5-HT system as a potential target for the preceding effects of binge eating during dieting. Specifically, they demonstrate decreased levels of cerebrospinal fluid 5-HIAA \cite{jimerson1992low}, less platelet binding of 5-HT uptake inhibitors \cite{marazziti1988involvement}, reduced availability of central transporters, and weaker neuroendocrine responses to substances that influence 5-HT levels such as meta-chlorophenyl piperazine (m-CPP) \cite{levitan1997hormonal}. Additionally, experiments involving tryptophan depletion, which lowers brain tryptophan and 5-HT synthesis, have been shown to worsen bulimic symptoms in active patients and temporarily trigger such symptoms in those who have fully recovered \cite{kaye2000effects}.

Hormonal Influences

The interplay between hormones and neurotransmitters, particularly the influence of leptin and ghrelin on dopamine and serotonin, is foundational to our understanding of appetite control and emotional well-being. Leptin, a hormone associated with satiety, and ghrelin, which triggers hunger, interact with dopamine and serotonin, neurotransmitters linked to reward and mood \cite{Klok2007RoleLeptinGhrelin}. This intricate connection suggests hormonal and neurotransmitter balances are critical for health and psychological well-being.

Leptin, produced by white adipose tissue, communicates with the brain and other parts of the body to regulate appetite and metabolism based on energy reserves. When adipocytes are adequately supplied with fat, they release leptin as a signal indicating sufficient energy reserves. The secretion of leptin escalates proportionally with elevated adipose reserves \cite{klok2006role}. Conversely, adipocytes reduce their leptin production as adipose reserves decline, signaling the brain to refill energy reserves and prompting hunger. Resistance to these hunger signals is interpreted by the brain as a state of starvation, leading the hypothalamus to decrease metabolism. Consequently, individuals undergoing dietary restriction may find themselves needing less food than before \cite{spalding2008dynamics}.

Consistent findings from studies examining changes in leptin levels among individuals with AN indicate a crucial role of leptin dysregulation in the disorder's pathophysiology. In these cases, hypoleptinemia (pathologically low levels of leptin) is evident, highlighting the disrupted energy balance characteristic of the disorder. This dysregulation contributes to the altered appetite regulation and metabolic disturbances observed in affected individuals \cite{hebebrand2003hyperactivity}. If female leptin levels decrease below a threshold of approximately two ug/l, it triggers a cascade of events that result in the down-regulation of the hypothalamic–pituitary–gonadal (HPG) axis, subsequently leading to amenorrhea \cite{ballauff1999serum}. Amenorrhea is another common symptom of AN, defined as the absence of menstruation during the reproductive years of a woman's life \cite{nawaz2023amenorrhea}.

Deficient leptin levels thus have a profound impact on fertility. The mechanism through which leptin influences fertility is multifaceted, primarily involving its role in regulating the HPG axis, which is central to reproductive health \cite{Vadakkadath2005}. When the leptin levels

sharply decrease, it signals energy deficiency, suppressing the HPG axis. This suppression results in reduced secretion of gonadotropin-releasing hormone (GnRH) from the hypothalamus, which consequently lowers the production of luteinizing hormone (LH) and follicle-stimulating hormone (FSH) by the pituitary gland. LH and FSH are critical for ovulation and spermatogenesis, and their deficiency leads to reduced fertility \cite{Holtkamp2003Reproductive}. The reduced secretion of LH and FSH directly affects the gonads. In females, this results in anovulation or irregular ovulation, leading to menstrual irregularities or amenorrhea and, thereby, reduced fertility. Reduced LH and FSH levels in males lead to decreased testosterone production and impaired spermatogenesis, affecting male fertility \cite{Wabitsch2001}.

Emerging evidence from leptin therapy studies in individuals with AN suggests that leptin restoration can enhance HPG axis function and thus improve fertility. Treatment with human recombinant leptin (metreleptin) elevates gonadotropins (LH and FSH), testosterone in males, and estradiol in females, indicating a return of reproductive function \cite{GradlDietsch2023MetreleptinAN}. This aligns with findings that leptin levels normalize with a healthy BMI range, implying its role in normalizing reproductive function. However, it is uncertain whether leptin alone is sufficient for menstrual function normalization or if it merely facilitates the process \cite{kopp1997low}. Audi et al. \cite{audi1998leptin} conducted a cross-sectional study involving 65 individuals with AN to investigate the correlation between leptin and hormonal imbalances. Additionally, they aimed to assess the potential impact of increasing leptin levels, either alone or in conjunction with other hormones, on the return of menstruation with weight gain. These patients were categorized into three groups: (I) underweight and amenorrheic; (II) weight-recovered but still amenorrheic; and (III) weight-recovered and menstruating women. Patients in group I had lower BMI, serum leptin, estradiol (E2), insulin-like growth factor 1 (IGF-1), and urinary growth hormone (GH) levels compared to those in groups II and III. Additionally, they exhibited higher sex hormone-binding globulin (SHBG) levels. While no differences in leptin levels or BMI were observed between amenorrheic and menstruating weight-recovered patients (groups II and III), free E2 and GH levels were significantly higher in weight-recovered individuals. The result suggests that while leptin plays a crucial role, it alone may not be adequate to trigger the resumption of menstruation in AN patients. Additional factors are likely involved in this process \cite{audi1998leptin}.

In contrast to AN, where the relationship between leptin levels and BMI is more predictable due to the low body fat content characteristic of the disorder, BN presents a more complex scenario. Despite often maintaining a normal or near-normal BMI, individuals with BN may exhibit altered leptin levels, suggesting that factors beyond mere adiposity influence leptin regulation.

Studies show that the metabolic consequences of erratic eating behaviors in patients with BN and psychological stress are implicated in the dysregulation of leptin levels. For example, Jimerson et al. \cite{Jimerson2000Decreased} identified decreased leptin function, which may be associated with alterations in eating patterns, metabolic rate, and neuroendocrine regulation in BN, proposing a connection between reduced leptin levels and the disorder's characteristic binge-purge cycle.

Jimerson et al. found notably reduced serum leptin levels in women with BN compared to healthy controls, even after adjusting for factors such as percent body fat. The finding suggests that the decreased leptin function in BN individuals might be attributable to factors beyond mere body composition differences \cite{Jimerson2000Decreased}. Significantly, the study uncovered a consistent pattern where lower leptin levels inversely correlated with the frequency of binge-eating episodes, implying that disruptions in leptin signaling, particularly within the hypothalamus, could underpin disordered eating behaviors by potentially diminishing satiety signals. This disruption may also interfere with serotonin pathways involved in satiety regulation, hinting at a complex interaction contributing to BN pathology \cite{Jimerson2000Decreased}.

In individuals with BN, reduced leptin levels can interfere with the body's natural mechanisms for controlling hunger and satiety, potentially exacerbating the binge eating and purging cycle \cite{Jimerson2000Decreased}. Moreover, this decrease in leptin functionality has broader implications on the neuroendocrine system, notably impacting metabolic processes and the overall hormonal equilibrium \cite{Obarzanek1991}. This kind of hormonal disturbance is believed to play a part in lowering thyroid hormone levels among BN sufferers and might disrupt the normal functioning of the hypothalamic-pituitary-gonadal axis \cite{Flier1998}. This axis's dysfunction underscores leptin's complex role beyond merely influencing body weight, highlighting its significant contribution to the broader neuroendocrine irregularities observed in BN \cite{Jimerson2000Decreased}. These observations point to the complicated link between leptin levels, metabolic rate, hormonal balance, and the regulation of the hypothalamic-pituitary-gonadal axis, emphasizing the hormone's critical influence on both the physical and psychological expressions of BN.

Significant attention has also been directed toward ghrelin, a peptide hormone predominantly produced by the stomach, in exploring endocrine influences on the pathogenesis of EDs. Ghrelin, often referred to as the "hunger hormone," is instrumental in regulating energy homeostasis, stimulating appetite, and promoting fat storage \cite{}. Its physiological role extends to modulating the gut-brain axis, influencing not only metabolic processes but also mood and stress responses, which are notably perturbed in EDs \cite{}. The literature outlines a complex and variable pattern of ghrelin levels across different ED diagnoses, suggesting a multifaceted involvement of this hormone in the etiology and maintenance of these conditions.

Given ghrelin's central function in signaling hunger to the brain, alterations in its circulating levels could contribute significantly to the disordered eating behaviors characteristic of AN, BN, and BED. For instance, elevated ghrelin concentrations have been documented in individuals with AN, potentially serving as a compensatory mechanism to stimulate food intake in the context of severe energy restriction \cite{}. Conversely, in conditions characterized by overeating, such as BED, ghrelin levels may be aberrantly modulated, reflecting a dysregulated appetite control system \cite{}.

Research by Fabbri et al.\cite{Fabbri2015} and Monteleone et al.\cite{Monteleone2005} provides essential insights into the role of ghrelin in EDs, revealing differing patterns of ghrelin levels across AN, BN, and BED. In the literature review conducted by Fabbri et al., 39 scholary articles published over a span of 14 years, from 2000 to 2014, were examined. Central to their findings was the observation of elevated ghrelin levels in individuals

diagnosed with AN, particularly within the subgroup engaging in binge/purge behaviours. The elevation may indicate the body's attempt to signal hunger and stimulate food intake in response to the extreme caloric restriction charecteristic of the disorder \cite{}. This hormonal response can be understood as a physiological effort to restore energy balance and mitigate the negative health impacts associated with prolonged undernutrition. A similar pattern was observed in BN patients, where a positive correlation was found between fasting ghrelin levels and the frequency of binge/purging episodes, proposing that ghrelin's dysregulation contributes to the cyclical nature of these disorders \cite{}.

Furthermore, Fabbri et al. delved into the genetic aspects of ghrelin by examining polymorphisms, variations in the DNA sequence that occur within a specific gene across different individuals \cite{}, associated with this hormone. These variations can influence how a gene functions, potentially affecting the production, secretion, and action of hormones such as ghrelin \cite{}. Fabbri et al. explored how different genetic polymorphisms within the ghrelin gene might predispose individuals to EDs by altering ghrelin's biological effects. These alterations can impact ghrelin's role in several physiological processes, including:

Appetite Regulation: ghrelin is often termed the "hunger hormone" because it stimulates appetite. Genetic polymorphsisms could lead to variations in ghrelin secretion or its ability to bind to receptors in the brain, thus influencing an individual's feeling of hunger and satiety \cite{}. For example, a polymorphism that results in higher ghrelin levels might increase appetite beyond normal levels, contributing to disordered eating patterns.

Energy Balance: Ghrelin plays a crucial role in managing energy balance by signaling the body to increase food intake and decrease energy expenditure when needed \cite{}. Polymorphisms affecting ghrelin's action could disrupt this balance, leading to abnormal eating behaviors or energy use, which are hallmark features of EDs.

Gastrointestinal Function: Beyond appetite regulation, ghrelin also affects gastrointestinal motility and secretion \cite{}. Changes in ghrelin's activity due to genetic variations might impact digestion and nutrient absorption, further complicating the relationship between food intake and body weight management in individuals with EDs.

Neuroendocrine Regulation: Ghrelin interacts with various neuroendocrine pathways, including those involving growth hormone release and the stress response \cite{}. Polymorphisms that alter these interactions could affect an individual's stress resilience, mood, and overall mental health, factors closely linked to the development and maintenance of EDs.

This study highlights the potential for developing targeted therapeutic interventions beyond traditional psychotherapy and nutritional counseling by examining how genetic variations influence ghrelin's functions, regulating appetite and satiety. Precisely, understanding these genetic factors can guide the development of pharmacological agents to modulate ghrelin secretion or block its receptor interactions, presenting novel treatment methods.

Moreover, identifying specific genetic polymorphisms that affect ghrelin's activity enables a more refined understanding of the etiological framework of EDs. This knowledge fosters the advancement of personalized medicine approaches, where interventions can be tailored to

address an individual's unique genetic profile, enhancing the efficacy and precision of treatment modalities. By focusing on the genetic variations that modulate the action of ghrelin, therapeutic strategies can be more directly aligned with the underlying biological disruptions present in individuals with EDs.

The practical implications of our genetic perspective are significant. It underscores the need to incorporate genetic testing into the diagnostic and treatment processes for EDs. This approach ensures that interventions not only aim to normalize ghrelin levels but also restore healthy eating behaviors in a manner customized to each patient's genetic makeup. By doing so, we significantly enhance the potential for successful outcomes, making it a crucial aspect of contemporary strategies in managing and treating EDs.

While the findings are promising, it is important to note that further research is needed to fully understand the complex interactions between ghrelin polymorphisms and EDs. Future studies should aim to expand on the initial findings, exploring a broader range of genetic variations and their impacts on different populations. This research is crucial to confirm the initial hypotheses, refine treatment approaches, and ultimately ensure that the potential of genetic insights is fully realized in clinical settings. This ongoing research will be instrumental in establishing more effective, personalized therapeutic strategies for EDs.
% https://www.scielo.br/j/rpc/a/cTQw45r9KKVMqm93sh6GXgy/?lang=en

The research by Monteleone et al. complements Fabbri et al. findings by investigating ghrelin levels in women diagnosed with BED. Their research focused on measuring plasma ghrelin levels in a group of women categorized into three distinct types: non-obese BED, obese BED, and obese women without binge eating behaviors, to understand the hormonal dynamics associated with this condition.

The study observed clear decreased plasma ghrelin levels in women with BED, both non-obese and obese, compared to control groups. This finding was consistent across BED patients regardless of obesity status, indicating that the reduction in ghrelin was directly associated with the disorder itself rather than BMI \cite{}.

Interestingly, the decrease in ghrelin levels was also noted in obese women who do not engage in binge eating, suggesting a potential commonality in ghrelin regulation among individuals with obesity, irrespective of their binge eating status. However, the reduction of ghrelin was more pronounced in those with BED, pointing to enhanced dysregulation in this group \cite{}.

The lower levels of ghrelin among BED patients is somewhat counterintuitive since ghrelin is typically elevated in states of energy balance (fasting or caloric deficit) to stimulate appetite \cite{}. Under normal conditions, ghrelin levels increase during fasting and decrease after meals, signaling hunger and satiety to the brain to regulate food intake. In BED, however, the expected increase in ghrelin levels in response to a perceived need for food intake does not occur, suggesting a disruption in the normal signaling pathway that might contribute to dysregulated eating behaviors \cite{}.

Monteleone et al. purpose that the reduction in ghrelin way represent an inadequate compensatory response aimed at counteracting the excessive caloric intake during binge

eating episodes. This implies that in BED, the body may attempt to reduce the drive to eat by suppressing ghrelin, but this mechanism fails to prevent binge eating episodes \cite{}. Highlighting the inadequate compensatory response of ghrelin suppression points to a potential disconnection or malfunction in the standard homeostatic mechanisms that regulate hunger and satiety. This insight opens up potential areas for therapeutic intervention,such as targeting ghrelin pathways or addressing the higher-order neural mechanisms that might be disrupting normal eating behaviors \cite{}.
%https://www.sciencedirect.com/science/article/pii/S0306453004001258?via%3Dihub

Treatment Approaches

Within the domain of ED treatment, psychotherapy is widely acknowledged for its importance, although there is limited literature dedicated to the biomedical aspect of therapy. Psychotherapy remains the primary approach, but the reluctance of patients to engage in therapeutic approaches presents notable challenges \cite{smink2012epidemiology}.

In addressing these challenges, it is essential to adopt an interdisciplinary approach. Optimally, an effective treatment team should consist of a medical doctor, psychiatrist, dietitian, and therapist working together, involving the patient's family and support network \cite{apa1994dsm}.

The biomedical treatment methods differ depending on the classification of EDs. For AN, psychotropic medications are discouraged as the exclusive or primary form of treatment according to American Psychiatric Association (APA) guidelines. However, they may be considered for individuals who have successfully restored their weight to help maintain the improvement or/and to address any comorbid conditions such as depression or obsessive-compulsive disorder (OCD). Additionally, anxiolytic medication can help reduce eating-associated anxiety, and olanzapine is noted for its effectiveness in stimulating appetite and enhancing weight in severe cases \cite{apa1993practice}.

There is a more body of evidence supporting the utilization of pharmacotherapy in the management of BN. The efficacy of fluoxetine, for instance, in treating BN is supported by diverse case reports, systematic studies, and double-blind, randomized, placebo-controlled trials. Fluoxetine is an antidepressant and a selective serotonin reuptake inhibitor (SSRI), which increases the levels of serotonin within the brain \cite{fluoxetine_ncbi}. In a double-blind, placebo-controlled clinical trial \cite{fluoxetine_bulimia}, 387 patients were randomly assigned to either the fluoxetine or placebo group, receiving daily doses of either 20mg or 60mg for eight weeks. Administering the lower dose of fluoxetine decreased occurrences of binge eating and vomiting compared to the placebo group, showing reductions of 45\% versus 33\% for binge eating and 29\% versus 5\% for vomiting, respectively. Those given 60mg of fluoxetine experienced even more significant improvements, with a 67\% decrease in binge eating and a 56\% decrease in vomiting. Other types of antidepressants, such as desipramine and amitriptyline, have also been found to be effective in the treatment of BN \cite{hughes1986}\cite{mitchell1984}.

Similar findings have been reported for medical treatments of BED. SSRIs, appetite suppressants, and antiepileptics have all demonstrated effectiveness \cite{arnold2002fluoxetine} \cite{appolinario2003sibutramine}. One promising medication in

this regard is Topamax, an anticonvulsant topiramate. According to a study published in The American Journal of Psychiatry \cite{mcelroy2003topiramate}, Topamax significantly reduced binge frequency and weight among individuals suffering from BED.

Severe ED conditions may necessitate additional measures. According to the Society for Adolescent Medicine \cite{golden2003eating}, one or more of the following criteria justify hospitalization:

1: Severe malnutrition (weight $\leq$75\% average body weight for age, sex, and height)

2: Dehydration

3: Electrolyte disturbances (hypokalemia, hyponatremia, hypophosphatemia)

4: Cardiac dysrhythmia

5: Physiological instability Severe bradycardia (heart rate < 50 beats/minute daytime; <45 beats/minute at night)

- 5.1 Hypotension (< 80/50 mm Hg)
- 5.2 Hypothermia (body temperature < 96° F)
- 5.3 Orthostatic changes in pulse (> 20 beats per minute) or blood pressure (>10 mm Hg)

6: Arrested growth and development

7: Failure of outpatient treatment

8: Acute food refusal

9: Uncontrollable binging and purging

10: Acute medical complications of malnutrition (e.g., syncope, seizures, cardiac failure, pancreatitis, etc.)

11: Acute psychiatric emergencies (e.g., suicidal ideation, acute psychosis)

12: Comorbid diagnosis that interferes with the treatment of the eating disorder (e.g., severe depression, obsessive compulsive disorder, severe family dysfunction)

Ensuring successful treatment of EDs necessitates that the patient remains hospitalized until their physical condition stabilizes, mental status improves, and a comprehensive care plan is established. A post-hospitalization study \cite{baran1995low} revealed that AN patients who achieved appropriate weight restoration (90–92% of ideal body weight) during hospitalization showed better overall outcomes than those who did not attain this goal. However, challenges within managed care systems often limit the length of hospital stays, leading to early discharge of patients while they are still underweight. This underscores the importance of ensuring sufficient duration for hospitalization to manage EDs effectively.

Cognitive Behavioral Therapy (CBT)

CBT stands as one of the most extensively researched and commonly implemented forms of psychological treatment. The core of cognitive therapy lies in gaining a clear understanding of one's thought processes, beliefs, and expectations. Its objective is to identify and modify erroneous and troublesome beliefs, recognizing that we often assign our perception and significance to events and situations that lead to problems, not necessarily the events themselves \cite{informedhealth}.

Unlike other therapeutic approaches such as psychoanalysis, CBT therapists prioritize understanding the individual's present circumstances over delving closely into their past experiences that may have contributed to their challenges. While a certain degree of historical information is necessary, the primary priority is on progressing to develop more efficient coping mechanisms for managing current life's demands \cite{apa-ptsd-guideline}.

There are various strategies involved in CBT treatment, including identifying and challenging distortions in thinking, gaining insight into others' behaviors and motivations, utilizing problem-solving techniques to navigate difficult situations, and fostering self-confidence. Additionally, CBT intervention targets behavioral patterns, aiming to bring about constructive changes. This aspect of treatment may involve confronting fears rather than avoiding them, engaging in role-playing exercises to prepare for complex interactions, and practicing relaxation techniques to achieve a calmer state of mind and body \cite{chand2023cognitive}.

When addressing EDs, the focus lies on aiding individuals in identifying and challenging their ingrained beliefs concerning self-image, body image, food, exercise, and self-worth. Therapy efforts to replace these destructive beliefs with more constructive and recovery-oriented viewpoints. For instance, individuals may be encouraged to reevaluate their perspective on self-worth, emphasizing internal qualities rather than external appearance, to promote a healthier relationship with themselves and their bodies. This approach involves supporting those struggling to recognize the beliefs perpetuating eating disorder behaviors and replacing them with more recovery-focused beliefs \cite{informedhealth}.

CBT-E and Eating Disorders

% https://www.ncbi.nlm.nih.gov/pmc/articles/PMC2928448/

CBT-E, an enhanced version of CBT, has emerged as a leading method for treating EDs and has been developed to improve treatment outcomes in this domain. It is tailored to address all forms of EDs, based on the concept that the factors involved in the persistence of these disorders share commonalities across various diagnoses rather than being unique to each one \cite{dejong2018enhanced}. This makes CBT-E a transdiagnostic treatment, meaning it targets a psychological process assumed to contribute to the development or perpetuation of multiple separate diagnoses.

CBT-E comprises four stages. In stage one, the primary focus is establishing a shared understanding of the patient's challenges related to food and eating. A helpful approach is to have the patient complete various questionnaires and maintain a self-monitoring journal of their eating behaviors and emotions associated with meals \cite{murphy2010cognitive}. The objective is to assist the patient and the therapist in identifying specific issues and evaluating the severity of the challenges while encouraging healthy eating habits and regular meal consumption throughout the day. A crucial aspect of this stage involves establishing patient-centric goals, enabling patients to assess their progress throughout therapy \cite{murphy2010cognitive}.

Building upon the groundwork laid in the previous stage, the therapist and patient in stage two engage in a collaborative assessment of progress. The primary objective is pinpointing remaining challenges and potential obstacles hindering further improvement

\cite{murphy2010cognitive}. This phase also allows for the reevaluation and possible adjustment of the treatment formulation as needed. The evaluation process fulfills various functions: patients making positive strides are acknowledged and encouraged, while those facing difficulties receive targeted attention to understand and address underlying issues. Adopting a more comprehensive treatment approach may be justified if clinical perfectionism, core low self-esteem, or interpersonal challenges emerge \cite{murphy2010cognitive}.

In stage three, the focus shifts to identifying and addressing the persistent factors contributing to ongoing struggles with food and eating habits. This tailored phase delves into critical aspects like managing concerns related to body shape and weight, enhancing coping mechanisms for daily challenges, and ensuring a balanced life where the preoccupation with food doesn't overshadow other important life domains \cite{murphy2010cognitive}. It also involves tackling extreme dietary restrictions and, if necessary, addressing issues like low self-esteem, perfectionism, or interpersonal difficulties that obstruct the path to overcoming eating disorders \cite{murphy2010cognitive}.

In the last stage, emphasis is placed on solidifying the improvements achieved and strategizing for sustained wellness. This concluding phase centers around crafting a comprehensive plan, in collaboration with the therapist, to mitigate the risk of relapse \cite{murphy2010cognitive}. This plan may incorporate reflections on past experiences that have shaped current destructive beliefs to rectify these foundational misconceptions. Additionally, there is an opportunity to acquire skills for better recognizing, managing, and expressing emotions, particularly those that have historically posed challenges, thereby reinforcing the journey toward lasting recovery from EDs \cite{murphy2010cognitive}.

Conversational Agents and Nutrition Technologies

Conversational agents, commonly known as chatbots, are sophisticated software programs designed to simulate human-like conversations with users through text or voice interactions \cite{TudorCar2020}. These virtual assistents utilize artificial intelligence (AI) technologies, such as natural language processing (NLP) and machine learning, to comprehend, analyze, and reply to user questions in a natural and user-friendly manner. The primary goal of conversational agents is to provide users with information, support, and services in a conversational format that mimics human dialogue, making interactions more engaging and accessible \cite{Saeed2016}.

In health and wellness settings, conversational agents have quickly become known for their ability to significantly change the way services are delivered and patient care is provided. They are utilized across various applications, including providing health information and guidance, aiding in symptom assessment and triage, enhancing patient education, and supporting disease management and mental health support \cite{MilneIves2020AIHealthCare}. These AI-driven assistants can offer 24/7 support, ensuring that individuals have direct access to health-related information and guidance whenever they need it, without the constraints of traditional healthcare delivery models.

The use of conversational agents in healthcare aims to enhance patient engagement, improve access to care, and support self-management of health conditions. By delivering

personalized and interactive health interventions, chatbots can facilitate improved connectivity between healthcare providers and patients, making healthcare more accessible and tailored to individual needs \cite{Laranjo2018}. Furthermore, they can lessen the strain on healthcare systems by automating routine questions and tasks, allowing healthcare professionals to focus on more complex patient care activities \cite{MilneIves2020AIHealthCare}.

One notable study is the randomized controlled trial by Fitzpatrick et al.\cite{Fitzpatrick2017Woebot}, which examined the effectiveness of Woebot, a fully automated conversational agent designed to deliver CBT-based self-help for young adults experiencing symptoms of depression and anxiety. Engineered to function via a messaging app interface, Woebot provided an engaging and intuitive platform for users, simulating therapeutic conversations by leveraging Natural Language Processing (NLP) to comprehend and react to user inputs in real time. In this study, 70 participants aged 18-28 were divided into two groups for comparative analysis: 34 individuals actively engaged with Woebot, while the other 36 were granted access to a digital CBT ebook \cite{Fitzpatrick2017Woebot}. This division enabled the researchers to assess the effectiveness of interactive, conversational support against a more traditional, static form of digital self-help content, thereby evaluating Woebot's unique contribution to managing symptoms of depression and anxiety in a young adult population.

The Woebot platform was developed to be highly accessible, leveraging the wide-spread use of messaging apps to ensure users' access comfort. Using text messaging, a platform familiar to many young adults, Woebot significantly reduced obstacles for those seeking mental health support \cite{Fitzpatrick2017Woebot}. This strategic choice of platform meant that users could interact with Woebot in a manner that felt both intuitive and convenient, facilitating regular engagement with the therapeutic tool. Its focus on interactivity was central to Woebot's design, allowing for dynamic conversations that closely mimic human interaction. This approach made the experience more engaging for users and facilitated the delivery of therapeutic content in a relatable and easy-to-understand manner \cite{Fitzpatrick2017Woebot}. The interactive nature of Woebot encouraged users to actively participate in their mental health care, engaging them in a two-way dialogue rather than passively consuming information.

Personalization was another key aspect of Woebot's functionality. The conversational agent utilized the responses provided by users to tailor subsequent content and conversations to their specific needs and preferences. This level of customization ensured that the support offered by Woebot was relevant to each individual's unique situation, enhancing the effectiveness of the intervention \cite{Fitzpatrick2017Woebot}. By adapting its interactions based on user input, Woebot created a more personalized and supportive experience critical in mental health care, where individual experiences vary widely.

The therapeutic content delivered by Woebot was grounded in CBT principles, one of the most widely supported therapeutic approaches for addressing depression and anxiety \cite{informedhealth}. Woebot's conversations and activities were designed to help users identify and challenge negative thought patterns, a core component of CBT \cite{informedhealth}. Additionally, Woebot encouraged users to engage in behavioral

changes that could lead to improved mental health, such as practicing gratitude or altering their routines \cite{Fitzpatrick2017Woebot}.

Furthermore, Woebot introduced users to the concept of cognitive distortions, which are irrational or exaggerated thought patterns that can contribute to feelings of anxiety and depression \cite{Shickel2020AutomaticDetection}. Through interactive dialogues, Woebot guided users in recognizing these patterns in their own thinking. For instance, if a user expressed feeling like a failure, Woebot might ask them to consider the evidence supporting and contradicting this belief, thereby illustrating the concept of "all-or-nothing" thinking \cite{Fitzpatrick2017Woebot}. This exercise encouraged users to step back and analyze their thoughts more objectively, helping them to identify distortions such as overgeneralization, catastrophizing, or personalization .

Building on the identification of cognitive distortions, Woebot then guided users through the process of challenging and reframing these negative thoughts. This involved asking users to consider alternative interpretations of situations that evoked negative emotions \cite{Fitzpatrick2017Woebot}. For example, Woebot might prompt a user to explore explanations for a friend's unresponsiveness other than the user's initial assumption that the friend was upset with them. Through this process, users learned to question the accuracy of their negative thoughts and to replace them with more balanced and realistic perspectives, a technique known as cognitive restructuring \cite{Larsson2016CognitiveTechniques}.

Gratitude practices were another critical element of Woebot's CBT-based exercises. These practices involved encouraging users to reflect on and enumerate aspects of their lives for which they were thankful \cite{Fitzpatrick2017Woebot}. Woebot might, for example, ask users to list three things that went well that day or to recall a recent experience that brought them joy. This exercise aimed to shift the user's focus from negative preoccupations and worries to positive experiences and emotions, fostering a greater sense of well-being and reducing symptoms of depression and anxiety \cite{Fitzpatrick2017Woebot}.

Through these CBT-based exercises, Woebot facilitated a dynamic and interactive learning process, enabling users to develop critical skills for managing their mental health \cite{Fitzpatrick2017Woebot}. The conversational agent's approach was characterized by gradual skill building, starting with identifying problematic thought patterns, moving through the critical evaluation and restructuring of these thoughts, and ultimately fostering a more positive outlook through the practice of gratitude. This structured yet flexible approach ensured that users could apply CBT principles to their daily lives, promoting lasting changes in their thought processes and emotional responses.

In the study conducted by Fitzpatrick et al. \cite{Fitzpatrick2017Woebot}, the findings revealed that participants who interacted with Woebot experienced notable decreases in depression and anxiety symptoms, with the group using Woebot demonstrating a statistically notable reduction compared to the group provided with a digital CBT ebook. Specifically, participants engaging with Woebot exhibited a mean decrease in depression scores measured by the PHQ-9 (Patient Health Questionnaire-9), indicating a substantial improvement in their mental health status \cite{Fitzpatrick2017Woebot}. This significant difference highlights the potential of conversational agents like Woebot in offering accessible and user-friendly mental health support. The ease of use and constant availability of Woebot

were highlighted as key factors contributing to its positive reception among users, supporting the viability of chatbots as practical tools for mental health care provision.
%As the technology behind conversational agents continues to evolve, their application in healthcare and wellness is expected to expand, offering new opportunities for improving health outcomes and patient experiences. However, the development and deployment of these tools in healthcare settings also present important considerations regarding their effectiveness, safety, and health information privacy.

Role of Nutrition Technologies

Following the exploration of conversational agents in mental health support, another considerable advancement in digital health interventions is the use of nutrition technologies. These technologies, ranging from mobile apps to comprehensive web-based platforms, are designed to help individuals overcome eating challenges by promoting healthy eating behaviors, offering nutritional education, and aiding in the management of disordered eating patterns \cite{Gabrielli2017MobileAppNutrition}.

In a comprehensive review by Olson \cite{Olson2016Behavioral}, the effectiveness of electronic (e-health) and mobile (m-health) communication technologies in behavioral nutrition interventions is thoroughly examined. The review analyzes studies from 2005 to 2009, demonstrating the impactful role of e-health technologies in fostering dietary changes among participants. Notably, these changes include a marked reduction in fat intake and a substantial increase in the consumption of fruits and vegetables, with approximately 75\% of the trials reporting positive outcomes \cite{Olson2016Behavioral}. Such dietary improvements are pivotal, with reducing fat intake playing a crucial function in improving heart health and lowering the risk of chronic diseases associated with high-fat consumption, such as cardiovascular diseases and obesity \cite{RuizNunez2016SaturatedFattyAcids}. Simultaneously, the increased intake of fruits and vegetables among participants highlights a preventive strategy against various chronic conditions, including heart disease, hypertension, certain cancers, and diabetes, due to the essential nutrients these foods provide \cite{Volpe2019FruitVegetable}.

Beyond these dietary improvements, the interventions have progressively embraced personalization, incorporating features like weight and behavioral self-monitoring. This shift towards personalized interventions has led to higher engagement levels among participants, with customized feedback and interventions tailored to individual needs significantly enhancing motivation \cite{Olson2016Behavioral}. This, in turn, facilitates more effortless and more sustainable healthful dietary changes. Moreover, integrating behavioral self-monitoring tools within these interventions has empowered participants to track their nutritional habits more effectively. Such real-time monitoring and feedback mechanisms heighten awareness of eating patterns, aiding in identifying areas for improvement and reinforcing positive dietary behaviors \cite{Olson2016Behavioral}.

Applications in Eating Disorders

Conversational agents and nutrition technologies are increasingly tailored to support individuals with EDs by employing various strategies to motivate healthier eating behaviors. These technologies use goal setting, feedback, personalized communication, and motivational interviewing techniques to encourage positive changes in eating habits.

A study by Kramer et al. \cite{Kramer2020HealthyLifestyleCoaching} provides a comprehensive overview of how Embodied Conversational Agents (ECAs) are designed and utilized to promote a healthy lifestyle. In their scoping review, they explore the current practices in designing ECAs, the applied behavior change techniques, the underlying theories, and the overall effectiveness of these agents.

Behavior change techniques (BCTs) are systematic procedures incorporated into an intervention to change behavior \cite{Michie2013BehaviorChangeTechnique}. In the context of ECAs for promoting a healthy lifestyle, these techniques could include goal setting, self-monitoring, providing feedback on performance, social support, and motivational interviewing. The use of these techniques is to engage the user actively, encourage commitment to health-related goals, and facilitate long-term behavior change \cite{Kramer2020HealthyLifestyleCoaching}. The theories behind these techniques often stem from psychological and behavioral science. For example:

Social Cognitive Theory (SCT) emphasizes observational learning, social experience, and reciprocal determinism in behavior change \cite{Gauthier2022Bandura}. For individuals with EDs, conversational agents can model healthy behaviors, such as mindful eating or positive body image, provide encouragement, and create a learning environment that reflects these principles. By observing modeled behaviors, users can learn to replicate them, potentially improving their relationship with food and body image.

Theory of Planned Behavior (TPB) suggests that intention is a direct influencer of behavior, affected by attitudes toward the behavior, subjective norms, and perceived behavioral control \cite{Ajzen1991TheoryPlannedBehavior}. In the context of EDs, conversational agents can be designed to positively influence these factors by providing information that shapes more positive attitudes toward healthy eating, normalizing recovery behaviors within social contexts, and enhancing the user’s confidence in their ability to make these changes. This approach can help create a supportive environment that encourages individuals to engage in healthier eating behaviors.

Self-Determination Theory (SDT) focuses on fulfilling the psychological needs of autonomy, competence, and relatedness to drive intrinsic motivation \cite{Ryan2000SelfDetermination}. For individuals with EDs, nutrition technologies that adhere to SDT principles can foster a sense of ownership over health goals, offer skill-building opportunities to manage eating habits healthily and create a supportive relationship that resonates with the user's needs for connection and understanding \cite{Matusitz2013The}. Individualizing interventions based on the user's specific challenges and progress can further enhance their engagement and motivation toward recovery.

The study discusses that successful ECAs incorporate multiple BCTs and grounding theories to ensure that interventions are theoretically valid, practical, and engaging for the user \cite{Kramer2020HealthyLifestyleCoaching}. For example, an ECA designed to promote physical activity might set personalized goals for the user, track their progress, and provide feedback and encouragement. This could be underpinned by theories that recognize the importance of personal motivation, social support, and the user's belief in their ability to achieve their goals.
