# Supplementary material for: Exploring Body Image Awareness With a Large Language Model–Based Conversational Agent: Qualitative Study With Young Adults
Source: J Med Internet Res. 2025 Nov 17;27:e78829. doi: 10.2196/78829 (PMC12670058; doi:10.2196/78829)
Supplement: Multimedia Appendix 3 [file jmir_v27i1e78829_app3.pdf]

TrueBalance’s Individual Interview

Table of contents

Pre-Interview *2*

1 Informed Verbal Consent 2

2 Introduction about TrueBalance 2

3 Pre-intervention Questionnaires 2

4 Introductory Questions 3

5 Theme Questions 3

1) Theme Questions: Perceived Health 3
2) Theme Questions: Understanding of Eating Disorders and Emotional Eating 3
3) Theme Questions: Body Imagine 3
4) Theme Questions: Technology Use in Health 4

6 Final Questions 4

Post-Interview *5*

1 Theme Questions 5

Theme Questions: Perceived Health 5
Theme Questions: Understanding of Eating Disorders and Emotional Eating 5
Theme Questions: Body Image 5
Theme Questions: User Experience with the Agent 6

2 Final questions 6

Xuan Zhang, 2024

Pre-Interview

1 Informed Verbal Consent

Hello, I'm Xuan Zhang, a researcher at KTH. Thank you for joining this project. Thank you for signing the consent form! Our study aims to develop a new conversational tool called TrueBalance, designed to promote positive body image and this is a user's study.

There will be two online meetings in total, during which you will complete questionnaires and participate in interviews with the researcher. This first meeting will last about 30 minutes, and the second, occurring 8-10 days later, will last 45-60 minutes. The entire study will tak
e approximately 2-3 hours.

2 Introduction about TrueBalance

We have developed a new tool called TrueBalance to offer an alternative to mainstream nutrition technologies. The goal of TrueBalance is to help people recognize and address dissatisfaction with their body image, which can lead to unhealthy eating habits. Currently, TrueBalance is still in the testing phase, so you can interact with the system through the following Telegram link: https://t.me/TrueBalanceBot?start=w29590311

TrueBalance allows users to have private, text-based conversations about topics like physical appearance, eating habits, fear of gaining weight, or food-related anxiety. The tool integrates medical factors that may contribute to eating disorders and uses principles of Cognitive Behavioural Therapy to help users identify negative thoughts and replace them with more positive, constructive thinking.

Meanwhile, TrueBalance is able to offer suggestions based on scientific knowledge about eating disorders, always keeping the user's well-being in mind. Importantly, it does not collect any personal information, such as names or identifying details. The all conversations remain completely anonymous.

We hope TrueBalance can help promote a positive body image, prevent eating disorders, or be used alongside professional therapy as additional support.

3 Pre-intervention Questionnaires

Please fill out this questionnaire now. Your participant ID is P1. Please use this ID when filling out the survey below. You will be asked about your body image satisfaction and social media use. This information will be important for research. Your answers will be anonymous. It will take around 10mins. The link is https://survey.kth.se/Survey/894

4 Introductory Questions

During this time, we'll discuss your current health status, your understanding of eating disorders and emotional eating, your body image, and your use of health-related technology.

I want to assure you that all your responses will remain completely anonymous, and your data will only be used for research purposes. Your comfort and privacy are our top priorities. If at any point during the interview, you feel uncomfortable or wish to stop, you're free to withdraw at any time without any consequences.

Before we dive into the main topics, I'd like to start with a few basic questions to get to know you a little better. These questions will help us understand some background information about you and your experience with technology.

a) How old are you?
b) How do you identify your gender?
c) What's your education level?
d) Do you use any conversational tools such as Google Assistant, Amazon Alexa, and Apple Siri? If so, which one have you used?

5 Theme Questions

1) Theme Questions: Perceived Health

Thank you for sharing a bit about yourself. Now, I'd like to ask a few questions about how you perceive your current health, both physically and mentally before using TrueBalance.

e) How would you describe your current physical health?
f) How would you describe your current mental well-being?

2) Theme Questions: Understanding of Eating Disorders and Emotional Eating

g) Have you ever heard of or looked into the terms 'emotional eating' or 'eating disorder' before? If yes, when did you first come across them? Where did you go for information, and what was that experience like?"
h) What do these terms mean to you, and how do you understand them?"
i) In your opinion, what factors do you think might lead someone to engage in emotional eating or develop an eating disorder?

3) Theme Questions: Body Imagine

j) What does "positive body image" mean to you?
k) What is your current level of satisfaction with your body now?
l) What is your current level of satisfaction with your appearance now?
m) Do you believe that positive body image is linked to mental well-being? In what way?
n) Are there any activities or habits that help you maintain a positive view of your body? What are they?

o) Do you ever feel like you want to change or modify your body? If so, when do you find yourself thinking about that?
p) Do you think raising awareness of body image issues among young adults is important?
q) From your perspective, what more could be done to improve awareness of body positivity among young adults?
r) Have you ever discussed these feelings with your friends or family members?

4) Theme Questions: Technology Use in Health

s) Have you used any technology to track your nutrition or physical activity? What type of technology?
t) Do you use social media? Which platforms do you use, and how often?
u) Do you think these technologies, like social media, affect your body image? If so, how have they influenced the way you feel about your body?
v) Have these technologies increased your desire to change your body or appearance? In what way?
w) How do you feel when I introduce TrueBalance to you?
x) In what way do you think TrueBalance will affect your health or well-being?
y) What is your expectation of TrueBalance compared to human beings?

6 Final Questions

z) Do you have any other question about this research?

Thank you so much for your time and for sharing your experiences with me. Your insights are incredibly valuable to our research. I am looking forward to talking to you again at the end of the week!

Post-Interview

Thank you so much for participating in the post-interview. In this session, we'll revisit some of the topics we discussed before, but now with a focus on how your thoughts or feelings may have changed after using TrueBalance. We'll start by talking about your perceptions of your physical and mental health, then move on to topics like eating disorders, emotional eating, body image, and finally, your experience with the TrueBalance agent.

1 Theme Questions

Theme Questions: Perceived Health

Thank you for sharing a bit about yourself. Now, I'd like to ask a few questions about how you perceive your current health, both physically and mentally, and whether using TrueBalance has influenced your thoughts on these areas.

a) Do you have any different opinions of your current physical health after using TrueBalance? If so, what are they? If not, what might be the reason?
b) Do you have any different opinions of your current mental well-being after using TrueBalance? If so, what are they? If not, what might be the reason?

Theme Questions: Understanding of Eating Disorders and Emotional Eating

Thank you for sharing your thoughts on your health. Now, let's move on to explore your understanding of eating disorders and emotional eating, and how TrueBalance may have influenced your perspective on these topics.

c) Do you know more about 'emotional eating' or 'eating disorder' after using TrueBalance? If yes, what was that experience like?"
d) After using TrueBalance, what factors do you think might lead someone to engage in emotional eating or develop an eating disorder?

Theme Questions: Body Image

Now that we've talked about your thoughts on your physical and mental health, let's focus more specifically on how you feel about your body and appearance. We're interested in learning about how TrueBalance might have influenced your body image.

e) What is your current level of satisfaction with your body after using TrueBalance?
f) What is your current level of satisfaction with your appearance after using TrueBalance?
g) What does "positive body image" mean to you after using TrueBalance? Has it changed? If so, how have your feelings about your body changed since using the system? If not, what might be the reason?
h) Do you ever feel the desire to modify or change your body after using TrueBalance? In what situations do you feel this way?
i) Were there any moments where you felt more aware of wanting to change your opinions of your body image while using TrueBalance?

j) After Using TrueBalance, are there any activities or habits that help you maintain a positive view of your body? What are they?
k) After using TrueBalance, did you notice any daily activities that make you feel less satisfied with your body? If so, what kind of activities?
l) After Using TrueBalance, do you believe that positive body image is linked to mental well-being? In what way?
m) After Using TrueBalance, do you think raising awareness of body image issues among young adults is important?
n) After using TrueBalance, what do you think could be done to better promote body positivity among young adults?
o) After Using TrueBalance, have you ever discussed these feelings with your friends or family members? Would you consider doing that?

Theme Questions: User Experience with the Agent

Thanks for sharing your thoughts on body image. Next, I'd like to explore your experience interacting with the TrueBalance agent. We're keen to understand how helpful you found the conversations and the advice provided.

p) What was your first impression when you started using TrueBalance?
q) Did the agent help you manage your feelings regarding body image? Why or why not?
r) Did the agent improve your awareness of your body image? Why or why not?
s) Do you think the agent provided useful advice? In what way?
t) Which aspects of the conversation with the agent did you find most beneficial?
u) Do you think the agent provided harmful advice? In what way?
v) Which aspects of the conversation with the agent did you find least beneficial?
w) I What do you think you got from using TrueBalance that you might not get from other support, like talking to doctors?
x) Would you consider continuing to use Truebalance?
y) Would you recommend your friends or families to use TrueBalance?
z) Do you have any advice on the service of Truebalance?

2 Final questions

Now that we've discussed your experience with the TrueBalance agent, I just have a few final questions to wrap up and make sure we've covered everything that's important to you.

aa) Is there anything else you would like to add about your experience?
bb) Thank you for answering all those questions. How do you feel after answering these questions?
cc) Would you be interested in the results of this study?

Thank you so much for your time and for sharing your insights—it's been incredibly valuable to our study. Before we finish, is there anything else you'd like to add or clarify? Once again, thank you for your participation, and I hope you have a great day!
