# Supplementary material for: Exploring Body Image Awareness With a Large Language Model–Based Conversational Agent: Qualitative Study With Young Adults
Source: J Med Internet Res. 2025 Nov 17;27:e78829. doi: 10.2196/78829 (PMC12670058; doi:10.2196/78829)
Supplement: Multimedia Appendix 4 [file jmir_v27i1e78829_app4.pdf]

TrueBalance's Pre-Interview Analysis

Xuan Zhang, 2025

Title	Theme	Category	Codes	Meaning unit
Enhancing Positive Body Image through Conversations with an AI Agent: Qualitative Study with Young Adults	Self-Perceived Health and Well-Being	Fluctuating Perceptions of Health	Stable health perception with occasional manageable concerns	P1:Yeah, I mean, I have good days and bad days where I feel less good about it. And then I have days where I feel really good about it. But for the most part, I feel neutral.
				P3: I would say neutral at most of the day. And I would say probably 50% are pleasant and the rest of it are just neutral. And I would probably have once a week that I feel upset. So yeah, that's the frequency.
				P4: That fluctuates as an interesting one. I think it's either neutral would be lower neutral, cuz I feel like that's, it depends on the day sometimes, you know, cuz sometimes out there gonna be like, oh, you know, I'm feeling good and you know, you get your stuff being so confident in some days it's like is like my skin is not doing great or feeling secure about another body part. And it's like I'm not feeling very confident.
				P9: Sometimes I felt less confident in my body and sometimes I feel more confident.
		Personal emotional connections to food	Comfort-seeking through food consumption	P1: And she had opened up to me that her mom had very similar behaviors and that they would often eat together as a comfort thing when they were struggling with something in life. So I think for her, it was a way of feeling connected to home and then also just feeling better when she's really negative feeling.
				P2: And I also gradually experience the emotional eating thing or the eating disorder. For example, I want to eat in a sweet food whenever I feel bad or sad or whenever I want to like celebrating or and I eat food, I consider food as a...Yes. How do you call that? Like when you want to calm yourself. Comfort food. And comfort things. For yourself dealing with stress.
				P6: Like I want more cakes or sweet food, sugars, an ice cream, this kind of food before the exam. But in normal situations, I think I don't need more. I don't need that kind of.
		The relationship between mental health and body image	Interplay between body image and mental healt	P1: My hundred percent exercise, like not overdoing it, but I go to Pilates once a week. And that's usually when I feel my best about how I look and how I function, because I feel strong, I feel like I accomplish something.
				P6: I think like if you're not happy person, your look will not be good. Yeah, if you're sad, you can't be. Regardless of beautiful girl or healthy girl. So I think mental health is really important only when you're energetic or happy, you will feel your physical body is better. I think.
				P7: The the the positive you will have a better emotion. If you pay attention to it. But most people can't ignore what they look like. So if you have the better looking, you will have better emotion and you will have the better mental health.
				P8: As I said, if your mind is okay, you can start working your body image. And sometimes it can be one will lead to the other. So it's like a cycle. Because if you look good on yourself, you feel much more, much better about yourself. You go
	Understanding of Eating Disorders and Emotional Eating	Eating out of control as a strategy to cope with negative feelings	Extreme eating habits triggered by stress or sadness	P1: And it would be when she was stressed and felt out of control of her life then, or things were chaotic or she was struggling with grief at one point, like her grandpa was not doing very well health-wise.
				P3: I think I know less about that term because I think eating disorder is like abnormal eating habits out of your usual trend. Yeah, so that's it.
				P4: I don't know the intricacies of it, but I have a general idea. Just eating when you stress or you like depressed or just something that like an emotional trigger and then you sort of way to comfort yourself is to eat it.
				P5: Yeah, so I think it's, yeah, from my understanding, like when you feel bad about something or sad or you're stressed emotionally in a way, so to feel better, you end up maybe eating something but without really being hungry, but just to feel avoid in a way. Yeah, so that's how I understand it.
				P6: So I think this, that was the first time I heard emotional eating. And yeah, like you will eat more before like some stress for events.
				P8: Yes, some people when they feel stressed, they start eating a lot. And then sometimes what they eat, if it was vegetables and fruits, you would be good. It's wouldn't be that bad, but it's like everything, junk food and lots of chocolate, which
	Body Image Perception	The understanding of positive Body Image	Self-acceptance and self-appreciation	P1: Well, ideally to me, if I were to say that I had positive body image, I think that for me, that would mean I would have neutral body image, which is something that I feel like cuz I feel like if you associate feeling good about your body or your about body bringing you good feelings, if it changes, then that can be distressing, even if it changes in a good way or a bad way. Like as in if it fits more into societal standards or further away from societal standards.
				P2: I think a positive body image will be somebody that kind of more related to something like a mental factor, like you confident within it, look healthy, it would showcase on the skin and the body and the ability to be a weapon and stress. Yeah, positive body image.
				P3: I'm content with my current status and I wouldn't be affected by my body's changes emotionally or physically. I wouldn't get affected by it. So that's my identify identification for it.
				P5: Okay, so let me think. You see yourself in the mirror and you feel nice about it. Like you don't judge yourself and you accept what you see. Not just accept, but you like it and also you respect it in a way.
			Valuing body functionality	P1: And I think to me specifically, it's I'm satisfied with how my body functions rather than how my body looks. And that to me is good body image.
				P2: I concern more about my health and I try to eat, yeah, I just thinking like I try to eat more quality food for my body so it can function well and stay healthy.
				P5: Whereas I think the focus should be on you cuz this is your body and you'll have to live with it for the rest of your life. So you should focus on it being healthy and respecting it a lot. So yeah, I wish I'd known that sooner.
		Body Image Insecurities	Higher self-expectation	P3: I feeling myself, not confidence about themselves of the body image and feeling discourage when seeing others are having maybe a better body shapes, a better routine.
				P6: But yeah, I remember I there's only once I'm so jealous or admired other people's. Like my friends, they're all very tall because I'm in the north part of China. Yeah, Shandong province, you know, everyone is so tall.
			body comparison with surroundings	P5: I have to say, I kind of compared myself with her at that point. Like, oh, why she? But maybe it's very important to have a very good body weight because she like she take care of it so much.
				P9: Sometimes when you compare yourself to others, you can find things that you're less satisfied with. And so of course, it has gone up and down through time.
		Influence of Cultural and Societal Standards	Regional differences in body perception	P6: I think it's the phenomenon in social media, especially around the Asian background, yeah, a lot of girls still dining for the skinny body.
				P1: I think the best I've ever felt about my body was going to a Swedish Sona. And obviously, like you're not wearing any clothes with anyone around you and just seeing how nobody cared. It wasn't, no one was trying to oppose or anything.
		The paradoxical influence of social media	Unrealistic beauty standards	P4: What kind of people you're calling, but the people that I follow up Instagram, like they obviously, cuz I'm really into fashion, right? So it's people who have really great aesthetics and great bodies and create skin and stuff. So like after
				P7: I want to be more saying that maybe because the and culture want the girl have the same and tall and the beauty level, not the body. But I know it's not the very, a very nice standard, but I still want to be more thin.
				P6: Like from the influencers. Yeah, if they start to show the healthy body, what could be a healthy body, I think it's very easily to influence the young adult. Yeah, because if all the influencers show them the skinny body is the most healthy
			Social media-induced comparisons	P9: Yeah, in a negative way, I would say. Cuz it's a lot of comparing. Yeah, it's, it triggers that comparison. I think social media.
				P1: Sometimes I think that if I'm looking for like outset outfit inspiration from fashion influencers, I sometimes rarely find myself thinking, oh, well, if I had their body type that would look good on me, but it wouldn't look good on me because I
			Educational potential of social media	P2: I think the best ways nowadays, young adults using the social media along social socials. So the social media will be a nice to and a platform to get to them and by spreading the positivity message about loving everybody. Yeah, and I also
				P4: Maybe make it something that can be done on like Instagram in a way that's alluring. I just, the younger age demographic would be positive. Like, I know, a quick questioning cuz like people don't like doing wrong surveys and
				P5: So yeah, I would say mostly negative, but I at the same time, I think it they're really good platforms to get expose to other variety of like bodies and stuff. So that's good body face and like everything. So yeah, but I really think it has to do
				P6: I think social media is very important. The phenomena here, it's very important because the young adults is very easily to be effective. So come from, start from the social media more. Yeah. Give them some courses. I'm not sure. It's not,
		Body Image Development Across Life Stages	Developmental influence on self-perception	P2: Yeah, I think it's so crucial. I think it's really a fraud. I think everybody, each of us will in some certain moment in outline from the teenager, especially from teenagers, even childhood to in teenagers to young adults, we will also like be
				P8: When I was a kid, I might have felt more like, oh, I'm just skinny and that I wanted more weight, but I could never achieve is of gaining weight. I don't know what it is in my life, but I think now it doesn't matter much. I still want to get
			Recognition of personal boundaries and detachment from external validation	P3: Just for me, I sometimes feel discouraged when I'm bringing these things up to with them because in his mind, I think he would expect me to be skinnier to some context, but I might feel that wrong. But then I realize I don't need to live up
				P4: I don't know how to say this in an eloquent way, but it's really not that deep. Like, y'all people care about what should affect, but who cares what people think. The people who are most important are the ones that you surrounds yourself
		Strategies for improving body positivity	Social support networks	P1: And so if I'm having a bad body day and I'm feeling like I'm overweight or I'm feeling like I don't look very good, I'll text my friends.
				P4: But I think friendships are helpful in that area. Because even if your friends are like, which I would say not most of the time, it just try to make you feel bad. I mean, like, no, you look great. No, you beautiful. And that's just how, if we
				P8: Yes, there were times that we were wanting to exercise and we were making like some challenges for each other. I think challenges is also something good because everyone WA likes a challenge. And sometimes challenges are something
			Exercise and physical well-being	P1: My hundred percent exercise, like not overdoing it, but I go to Pilates once a week. And that's usually when I feel my best about how I look and how I function, because I feel strong, I feel like I accomplish something. And even though
				P6: I think, yeah, gym is really good choice. And also, I think doing some jogging, I mean, running around the park, this also a good way to embrace the nature and feel good about yourself. Yeah, especially when you go out with your friends.
				P8: Maybe doing sports, group activities. I think people were depending on the groups. It might not be lucky, but I think often the people will try to help each other out and create a good environment and that we'll try to maybe motivate you
				P9: But I started like working out more and that made me feel more confident in my body and not because of like, I'm not try to shave like weight loss or something like that to just, it's, we feel confident the way that I feel strong hum and
			Recognizing distorted reality	P9: So you kind of get disconnected from reality and how you perceive your body. Yes. And not so very truthful way.
				P1: Yeah, and a lot of people like edit their waists and do filters on their videos and you don't you can't even tell that they're on there. So then it's even more impossible to achieve.
			Positive self-talk for self-acceptance	P1: But I've also learn to just like tune out those thoughts and remind myself you're healthy, you're good, you take care of yourself and you have nothing to worry about. So that's where I'd say I am right now in terms of how I feel about my
				P9: And like, yeah, I always thinking when these kind of bad thoughts comes in and you try and you like self criticize yourself. I try to be kind. And for example, like again, if you go into social meeting, you see other people there, like always
			Use of makeup and cosmetic enhancements	P5: But yeah, those two would focus a lot on perfection and how your face should look amazing. And yeah, and it really helps him, helps them with their confidence when their face looks nice, like when their, yeah, makeup is great. But yeah, I don't have that.
				P7: But I still like to make up or dressing myself to make my appearance better.
			Social Media Detox	P4: Yeah, I know I try. Sometimes I stop using Instagram for like 3,4 months. So yeah, I can't, I'm really aware of like the inactive impact it can have. So I try to really try stay on top of that.
				P7: I think they can avoid to have too much social media, which is focus on like the lose weight or how to make up yourself, how to look you better and avoid this information on the social media and to focus, pay more attention on the reality
